# How reliable are MEG resting-state connectivity metrics?

**DOI:** 10.1016/j.neuroimage.2016.05.070

**Published:** 2016-09

**Authors:** G.L. Colclough, M.W. Woolrich, P.K. Tewarie, M.J. Brookes, A.J. Quinn, S.M. Smith

**Affiliations:** a Oxford Centre for Human Brain Activity (OHBA), University of Oxford, Oxford, UK; b Centre for the Functional Magnetic Resonance Imaging of the Brain (FMRIB), University of Oxford, Oxford, UK; c Dept. Engineering Sciences, University of Oxford, Parks Rd, Oxford, UK; d Sir Peter Mansfield Magnetic Resonance Centre, School of Physics and Astronomy, University of Nottingham, University Park, Nottingham, UK

**Keywords:** MEG, Source leakage, Magnetic field spread, Functional connectivity, Network analysis, Connectome

## Abstract

MEG offers dynamic and spectral resolution for resting-state connectivity which is unavailable in fMRI. However, there are a wide range of available network estimation methods for MEG, and little in the way of existing guidance on which ones to employ. In this technical note, we investigate the extent to which many popular measures of stationary connectivity are suitable for use in resting-state MEG, localising magnetic sources with a scalar beamformer. We use as empirical criteria that network measures for individual subjects should be repeatable, and that group-level connectivity estimation shows good reproducibility. Using publically-available data from the Human Connectome Project, we test the reliability of 12 network estimation techniques against these criteria. We find that the impact of magnetic field spread or spatial leakage artefact is profound, creates a major confound for many connectivity measures, and can artificially inflate measures of consistency. Among those robust to this effect, we find poor test-retest reliability in phase- or coherence-based metrics such as the phase lag index or the imaginary part of coherency. The most consistent methods for stationary connectivity estimation over all of our tests are simple amplitude envelope correlation and partial correlation measures.

## Highlights

• Comparison of the repeatability of 12 common network estimation methods.
• Consistency of estimation tested at group-level, subject-level and between subjects.
• Best-performing methods are correlations in band-limited power.
• Methods should correct for the effects of spatial leakage between sources.

## Introduction

Resting-state connectivity estimation with electrophysiology is an important tool for studying intrinsic brain activity. It is able to act in concert with resting-state functional magnetic resonance imaging (fMRI), both by deconstructing the oscillation-specific functional origin of resting-state networks (Brookes et al., 2011, Marzetti et al., 2013), and as a feasible tool for assessing the short-timescale fluctuations of connectivity at rest (Baker et al., 2014, de Pasquale et al., 2010, de Pasquale et al., 2015). It acts as a complementary modality to fMRI and electrocorticograms for analyses of cortical communication patterns (de Pasquale et al., 2015), as well as of the fundamental differences in connectivity between healthy and diseased populations (Stam and van Straaten, 2012, Stam, 2014, van Straaten and Stam, 2013).

There are some key challenges associated with connectivity modelling in magnetoencephalography (MEG). Firstly, the activity of the cortical sources responsible for the sensor recordings must be estimated (connectivity estimation between sensors being hard to interpret; Schoffelen2009, Schoffelen2009). Source estimates are highly spatially correlated, and this spatial leakage of inferred sources into their local neighbourhood can create the semblance of connectivity between regions that in truth just share components of the same sensor signal (Palva and Palva, 2012). Secondly, the relatively low rank of electrophysiological data places a limit on the number of independent network nodes that can be sensibly specified in a connectivity model (there is little merit in estimating a network matrix over a 2 mm source grid, for example). We can, however, attempt to describe as many of the dominant cortical sources as our data allow using sensible regions of interest (ROIs). Thirdly, the low signal-to-noise ratio (SNR) of MEG and EEG data creates noisy connectivity estimates: for example, there is less consensus about the spatial characterisation of conventional resting-state networks as expressed in MEG than there is in fMRI. Lastly (and this challenge is shared in fMRI modelling), it may be desired to estimate just the direct functional connections within the cortical network, or to assign directionality of information flow between nodes. Methods for achieving these goals, for example by partialling out data (Marrelec et al., 2006) or using temporal precedence as an indicator of causality (Granger, 1969, Baccala and Sameshima, 2001), are noisier to estimate, and the extent to which they show repeatable or reliable behaviour in MEG is poorly understood.

A large number of connectivity estimation methods are available to resting-state researchers. These may assess spectral coherence, the presence of a consistent phase delay, or the amplitude coupling of oscillatory patterns. Some of these methods estimate directionality, and some direct network connections. Not all authors use metrics which are insensitive to spatial leakage confounds, even after recent work highlighting the associated problems (Palva and Palva, 2012, Colclough et al., 2015, Brookes et al., 2012, Hipp et al., 2012, Maldjian et al., 2014). Despite this wide range of practice, the reliability and consistency of these network measures have not been fully compared. As a result, many researchers may be picking analysis techniques without an understanding of the reproducibility of their results. We seek to provide guidance to the researcher who is setting out on a resting-state analysis as to which network estimation method to choose.

We believe a key property that any network connectivity measure must satisfy is the repeatability and consistency in estimation of both the single-subject result and the group-level structure. Consistency of estimation is a measure of the noisiness of a network metric, given a set of resting-state scan protocols and source reconstruction methods. Indeed, the sensitivity of inference on the mean effect, by the reduction of noisy sources of variability, can be a good surrogate test in the absence of a known ground truth, even in the presence of smaller (and potentially informative) sources of variation in the network matrices (Smith et al., 2005). Knowledge of estimation consistency is also clinically relevant, as a key requirement in the development of normative population distributions. An understanding of the run-to-run variability of network estimation is a crucial first step when moving onward from group comparison studies to the classification or characterisation of new subjects.

In this article, we compare the consistency and reproducibility of whole-brain network estimation between 39 functionally-defined cortical ROIs both at the group-level, and within and between individual subjects for 12 commonly-employed network estimation metrics. We include in our analysis connectivity measures which are susceptible to spatial leakage confounds, not because they are necessarily to be recommended but because they are still used in the field. We will explore the extent to which the false connectivity induced by these confounds can artificially increase the consistency of network estimation. We concentrate on stationary connectivity, rather than characterising the evolution of networks over time in each subject. We perform our analysis on the resting-state MEG data provided by the Human Connectome Project (HCP), for a number of reasons. The data are freely available, have passed through a stringent pre-processing pipeline, include a large number of young, healthy subjects (61 with resting scans in the MEG2 release), and include three repeated sessions of resting-state recording for each participant.

As an alternative to the range of methods for connectivity estimation that are robust to spatial leakage artefacts, there are some source reconstruction approaches that are designed to minimise the cross-talk between specific cortical locations (for example, Hui et al. (2010), Hauk and Stenroos (2014), Dalal et al. (2006)). These methods require the multi-source reconstruction to have fewer dipoles than the rank of the sensor data: each ROI would have to be described by a single principle component of its lead fields or a representative voxel. The relative benefits of performing the dimensionality reduction down to the number of ROIs either before or after the source reconstruction (as we do in this paper) remain to be explored.

Other authors have essayed similar studies, but without the range of metrics presented here, or without the focus on source-space connectivity. Astolfi et al. (2007) found partial directed coherence (PDC; Baccala and Sameshima, 2001) to reliably infer connectivity in a Stroop task; Hardmeier et al. (2014) assessed the phase lag index (PLI; Stam et al. (2007)) and weighted phase lag index (wPLI; Vinck et al. (2011)) in the resting state, but only analysed sensor-level signals, not cortical sources; Wang et al. (2014) undertook a large simulation study to assess accuracy of network estimation, using the outputs from neural mass models and simulated fMRI data, but they did not look at reproducibility in real datasets; and Jin et al. (2011) and Deuker et al. (2009) both focussed on the reproducibility of graph metrics, without studying the underlying stationary connectivity estimates.

We present a detailed reproducibility analysis of commonly-used connectivity estimation methods, covering directed, undirected, partial and marginal techniques. We are not aiming to propose one individual measure as the best, but to determine, using real data, which of the measures in common use show sufficient reliability to provide a platform for inference of resting-state connectivity.

## Methods

### MEG data

Resting-state MEG recordings were collected on a whole-head Magnes 3600 scanner (4D Neuroimaging, San Diego, CA, USA) from 61 subjects as part of the HCP MEG2 release (Larson-Prior et al. (2013), Van Essen et al. (2013); 67 subjects were included in the release, but resting-state recordings that passed the quality control checks (which included tests for excessive SQUID jumps, sensible power spectra and correlations between sensors, and for sufficiently many well-behaved recording channels) were not available from six^1^). Of these, 22 are monozygotic twins, 22 are dizygotic twins and the remainder are unrelated. All subjects are young (22–35 years of age) and healthy. Resting-state measurements were taken in three consecutive sessions for each subject with little or no break in between, for 6 min each. The data have been provided pre-processed, after passing through a pipeline to remove any artefactual segments of time from the recordings, identify any recording channels that are faulty, and to regress out artefacts which appear as independent components in an ICA decomposition with clear artefactual temporal signatures (such as eye-blinks or cardiac interference). Sensor-space data were down-sampled from 509 Hz to 300 Hz to facilitate processing, with the application of a zero-phase anti-aliasing filter.

### Source localisation

MEG data from each session were source-reconstructed using a linearly constrained minimum variance scalar beamformer (Van Veen et al., 1997, Robinson and Vrba, 1999, Woolrich et al., 2011), which shows limited susceptibility to field spread (Schoffelen and Gross, 2009) and performs well even with correlated sources (Brookes et al., 2007). Pre-computed single-shell source models are provided by the HCP at multiple resolutions, registered into the standard co-ordinate space of the Montreal Neuroimaging Institute. Data were beamformed onto a 6 mm grid using normalised lead fields and estimates of the data covariance computed separately in the 1–30 Hz and 30–48 Hz bands. Covariance estimation was regularised using PCA rank reduction. The rank was conservatively reduced by five more than the number of ICA components removed during preprocessing (2–20 components, with a mean of 7). Source estimates were normalised by the power of the projected sensor noise. Source-space data were filtered into delta (1–4 Hz), theta (4–8 Hz), alpha (8–13 Hz), beta (13–30 Hz), low beta (13–20 Hz), high beta (20–30 Hz) and low gamma (30–48 Hz) bands.

### Parcellation

We were interested in comparing common network estimation methods for whole-brain, region-of-interest connectivity analysis. We used the parcellation of 39 ROIs employed in Colclough et al. (2015), which are clusters identified from a resting-state ICA decomposition of fMRI data from the first 200 subjects of the HCP. A single time-course was constructed to represent each node as the first principal component, after weighting the PCA over voxels by the strength of the ICA spatial map. This analysis yielded 39 time-courses for each frequency band and session.

### Removing contributions from source leakage

We compensate for spatial leakage confounds using a symmetric orthogonalisation procedure (Colclough et al., 2015) to remove all shared signal at zero lag between the network nodes. This procedure is a multivariate extension of the orthogonalisation principle proposed in Brookes et al. (2012) and Hipp et al. (2012), and allows for multivariate analyses (e.g. regularised partial correlation) to be straightforwardly carried out. It identifies the set of signals least displaced from the initial, uncorrected set, whilst enforcing mutual orthogonality between them, with no bias related to any reordering of the nodes. It removes any zero-lag signal overlaps that could be attributed to spatial leakage effects, before performing a multi-variate network analysis. There is evidence that that this methodology is imperfect for non-Gaussian data (Brookes et al., 2014). The approach was designed to correct correlation-based analyses on power envelopes for artefactual connections caused by the MEG point-spread function; in this work we test all metrics with and without this correction to understand the extent to which spatial leakage engenders artificial consistency in network estimation.

In the main text, we only apply this leakage correction to metrics that are sensitive to source leakage and do not have an equivalent corrected form. For completeness, results with this correction applied to all metrics are available in the supplementary information.

The orthogonalisation was applied in the time windows over which the network metrics were computed (O'Neill et al., 2015): for the correlation, spectral and autoregressive model measures, this was the entire time-series; for the phase-based metrics, it was applied within each epoch.

### Connectivity estimation

Connectivity between each region pair was subsequently estimated using the connectivity metrics described below. Generally these metrics fall into marginal (full) and partial network estimation measures, with directed and undirected edge orientation (see also Table 1).

#### Phase estimation methods

##### Phase lag index, weighted phase lag index and phase locking value

PLI, wPLI and PLV are all measures for assessing phase synchrony between two signals in a particular frequency band, formed from estimates of the instantaneous phase of the signal. In the task and resting-state literature, these measures are commonly computed in trials or artefact-free epochs of 2 s to 25 s, taking average values of these epochs (Vinck et al., 2011, Stam et al., 2007, Stam et al., 2009, Tewarie et al., 2014, Lachaux et al., 2000, Hardmeier et al., 2014). We used window lengths of both 2 and 10 s, with little or no change in the results: we therefore present data based on 10 s epochs (results from 2 s epochs are available in the supplementary information).

The analytic signal *h*(*t*) is derived from a measured signal *s*(*t*) as

(1) $$h=s+i\hat{s}=|z\left(t\right)|e^{\mathrm{i\phi}\left(t\right)},$$

where *ϕ* is the instantaneous phase and

(2) $$\hat{s}\left(t\right)=p.v.\int_{-\infty }^{\infty }\text{‍}\mathrm{d\tau}\;\frac{s\left(\tau \right)}{t-\tau }$$

is the Hilbert transform of signal *s* within each epoch (p. v. indicates the Cauchy principal value).

The phase-locking value (Lachaux et al., 1999) between two signals is given by

(3) $$\mathrm{PLV}=\left|\left\langle e^{\mathrm{i\Delta \phi}\left(t\right)}\right\rangle \right|,$$

where $\langle \dot{\rangle }$ indicates the expectation operator, over time and epochs.

The phase-lag index (Stam et al., 2007) quantifies the asymmetry of the phase difference, rendering it insensitive to shared signals at zero phase lag (see Section 2.4 above),

(4) $$\mathrm{PLI}=|\langle \text{sign sinΔϕ}\rangle |,$$

while the weighted PLI (Vinck et al., 2011) attempts to further weight the metric away from zero-lag contributions,

(5) $$\mathrm{wPLI}=\frac{\left|\langle z_{1}z_{2}\mathrm{sin\Delta \phi}\rangle \right|}{\langle |z_{1}z_{2}\mathrm{sin\Delta \phi}|\rangle }.$$

##### Mutual information of the phase

The mutual information shared in the instantaneous phases of two signals is a non-linear measure of their coupling, first proposed by Paluš (1997)

(6) $$I=\int_{-\pi }^{\pi }\text{‍}d\phi_{1}d\phi_{2}\;p\left(\phi_{1},\phi_{2}\right)\log\frac{p\left(\phi_{1},\phi_{2}\right)}{p\left(\phi_{1}\right)p\left(\phi_{2}\right)}.$$

As suggested in Wilmer et al. (2012), we normalise the mutual information by the joint entropy, *E*, to get a normalised mutual information estimand *ι* between 0 and 1,

(7) $$\iota =\frac{I}{E},\;E=-\int_{-\pi }^{\pi }\text{‍}d\phi_{1}d\phi_{2}\;p\left(\phi_{1},\phi_{2}\right)\log p\left(\phi_{1},\phi_{2}\right).$$

We used Peng et al.'s Matlab toolbox for computing the mutual information (Peng et al., 2005).

#### Spectral estimation methods

##### Coherence and imaginary coherence

The coherency of two signals is their normalised cross-spectral density,

(8) $${\tilde{C}}_{\mathrm{ij}}\left(f\right)=\frac{{\tilde{S}}_{\mathrm{ij}}\left(f\right)}{\sqrt{{\tilde{S}}_{\mathrm{ii}}\left(f\right){\tilde{S}}_{\mathrm{jj}}\left(f\right)}},\;{\tilde{S}}_{\mathrm{ij}}=\langle {\tilde{s}}_{i}\left(f\right){\tilde{s}}_{j}^{\text{★}}\left(f\right)\rangle ,$$

where the star indicates the complex conjugate. This provides a measure of the extent to which they share a constant relative phase. We estimated the cross-spectral density of each pair of ROI time-courses, for each session and frequency band, using Welch's method, with windows of length 10 s and a Hamming windowing function. We repeated this analysis (results in supplementary material), as for the phase-based metrics, using windows of 2 s, again finding little or no change in the results. We estimated the coherency for the complete time-series using these cross-spectra. Coherence, the absolute value of coherency, was averaged over the frequency band of interest.

The imaginary part of coherency (Nolte et al., 2004) is insensitive to phase synchronisation with no phase difference, and thus to the effects of signal leakage. Again, we computed coherency as above, and averaged the imaginary part of coherency over the frequency band of interest.

##### Partial coherence and imaginary partial coherence

The partial coherency of two signals is the normalised inverse of their cross-spectral density,

(9) $$\tilde{P}C_{\mathrm{ij}}\left(f\right)=\frac{{\tilde{S}}_{\mathrm{ij}}^{-1}\left(f\right)}{\sqrt{{\tilde{S}}_{\mathrm{ii}}^{-1}\left(f\right){\tilde{S}}_{\mathrm{jj}}^{-1}\left(f\right)}},$$

and is to coherency as partial correlation is to correlation: two signals are partially coherent if they retain coherency conditional on the spectra of all other signals in the sample. Partial coherence and partial imaginary coherency are obtained from the magnitude and imaginary part of partial coherency, respectively.

##### Phase slope index

The phase slope index (Nolte et al., 2008) is a directed phase synchronisation index which weights information from different frequencies. It is insensitive to leakage effects, and can handle non-linearities in the phase spectrum. It is defined as

(10) $$\mathrm{PS}I_{\mathrm{ij}}=ℑ\left(\sum_{f}{\tilde{C}}_{\mathrm{ij}}^{*}\left(f\right){\tilde{C}}_{\mathrm{ij}}\left(f+\mathrm{\delta f}\right)\right),$$

where I indicates the imaginary part and the summation is over frequencies *f* within the band and *δf* indicates the frequency resolution of the Fourier transform. We calculated PSI in the same epochs as PLI and PLV, averaging the results over the windows.

#### Auto-regressive models

##### Partial directed coherence

The Partial Directed Coherence (PDC; Baccala2001, Baccala2001) is a directed measure of signal transfer from one site to another. Modelling the source-space signals *X* as white noise passed through a complex transfer function *H* (normally inferred by fitting an auto-regressive model),

(11) $$\tilde{X}\left(f\right)=H\left(f\right)\tilde{E}\left(f\right),$$

where $\tilde{A}$ denotes the Fourier representation of time-domain signal A, the partial directed coherence is given by

$\mathrm{PD}C_{\mathrm{ij}}\left(f\right)=\frac{H_{\mathrm{ij}}^{-1}\left(f\right)}{\sqrt{\sum_{l}‍{H_{\mathrm{lj}}^{-1}}^{*}H_{\mathrm{lj}}^{-1}}}.$ (12)

PDC is often regarded as insensitive to source leakage, and has been defended as such (Kaminski and Blinowska, 2014), but other authors have since thrown doubt on this fact (Leistritz et al., 2013, Omidvarnia et al., 2014).

To compute this metric, we fitted an autoregressive model to the source-reconstructed signals in each ROI, for each band, by first downsampling to 2.5 times the Nyquist frequency of the band of interest, then fitting an AR model with 69 lags and computing the PDC and DTF using A. Schlögl's time series analysis toolbox.^2^ For the beta and gamma bands, this approach produced poor model fits and PDC estimations, but an AR model with 201 lags applied to the data in each of these bands, maintaining the sample rate at 300 Hz, produced good results.

#### Amplitude coupling methods

##### Amplitude envelope correlation and partial correlation

We estimated the amplitude coupling between ROIs using linear correlations and partial correlations of the envelopes of the band-pass filtered signals (Brookes et al., 2012, Hipp et al., 2012). Power envelopes were computed as the magnitude of the analytic signal, *h*, formed from the Hilbert transform of the ROI time-courses according to equation, low-pass filtered at 1 Hz and down-sampled to 0.5 Hz (Luckhoo et al., 2012). We then took the linear correlation between the logarithm of ROI power envelopes, and computed partial correlations by inverting the covariance matrix of the power envelopes, and regularising using the DP-glasso (Mazumder and Hastie, 2012). The regularisation parameter was chosen separately for each session and frequency band as that which maximised the corrected Akaike information criterion under 5-fold cross validation on the data within that session.

### Reliability and consistency of network measures

We assessed the network measures under test in three ways: (i) in their ability to produce consistent estimates of the population's connectivity patterns, (ii) to show consistent connectivity patterns between separate recording sessions of the same individuals, and (iii) similar connectivity between different subjects. Tests (i) and (iii) are closely related, being driven by the same sources of variation. After computing a network matrix for each recording session, estimates of group connectivity were made by taking the mean value at each network connection, over all sessions and subjects.

For the first test, we assessed the reliability of these group-level estimates by randomly dividing the dataset in half 100 times (keeping all sessions for each subject within a single half), computing group connectivity matrices with each network measure for each half, and correlating the edge strengths of these group matrices between each partition.

For the second test, reliability of network estimation within-subject was tested by correlating the edge strengths between network matrices generated from each of the three scans for each subject.

For the third, the consistency of networks estimated from different subjects was tested by correlating edge strengths between network matrices computed from different subjects.

We further investigated the relevance of individual edges to the creation of highly repeatable group-level metrics. On each edge, we calculated the contribution towards the correlation between the networks inferred from each half of the dataset. Thus, if the set of network edges inferred from one half of the data are denoted *x* and the other set *y*, the correlation between the two is

(13) $$\begin{array}{l} \rho =\frac{\sum_{i}\left(x_{i}-\langle x\rangle \right)\left(y_{i}-\langle y\rangle \right)}{\sqrt{\sum_{i}{\left(x_{i}-\langle x\rangle \right)}^{2}}\sqrt{\sum_{i}{\left(y_{i}-\langle y\rangle \right)}^{2}}}\\ \;=\sum_{i}z_{i}^{x}z_{i}^{y}, \end{array}$$

for $z^{x}=\frac{\left(x_{i}-\langle x\rangle \right)}{\sqrt{\sum_{i}‍{\left(x_{i}-\langle x\rangle \right)}^{2}}}$. The relative contributions from each edge, *i*, to our measure of reliability, *ρ*, are then given by the element-wise products $z_{i}^{x}z_{i}^{y}/\sum_{i}z_{i}^{x}z_{i}^{y}$.

Network matrix correlation scores were converted to *Z*-values using Fisher's transformation. This is a monotonic transformation which produces a clearer separation of results when correlations approach unity.

## Results

We focus on presenting results just from the alpha band in this section, because the differences between metrics were smallest for this band, presumably because it exhibits the largest signal to noise ratio. Other bands show exaggerated versions of the plots here, but follow the same patterns. They are presented in the supplementary information.

### Group connectivity structure

**Fig. 1:**
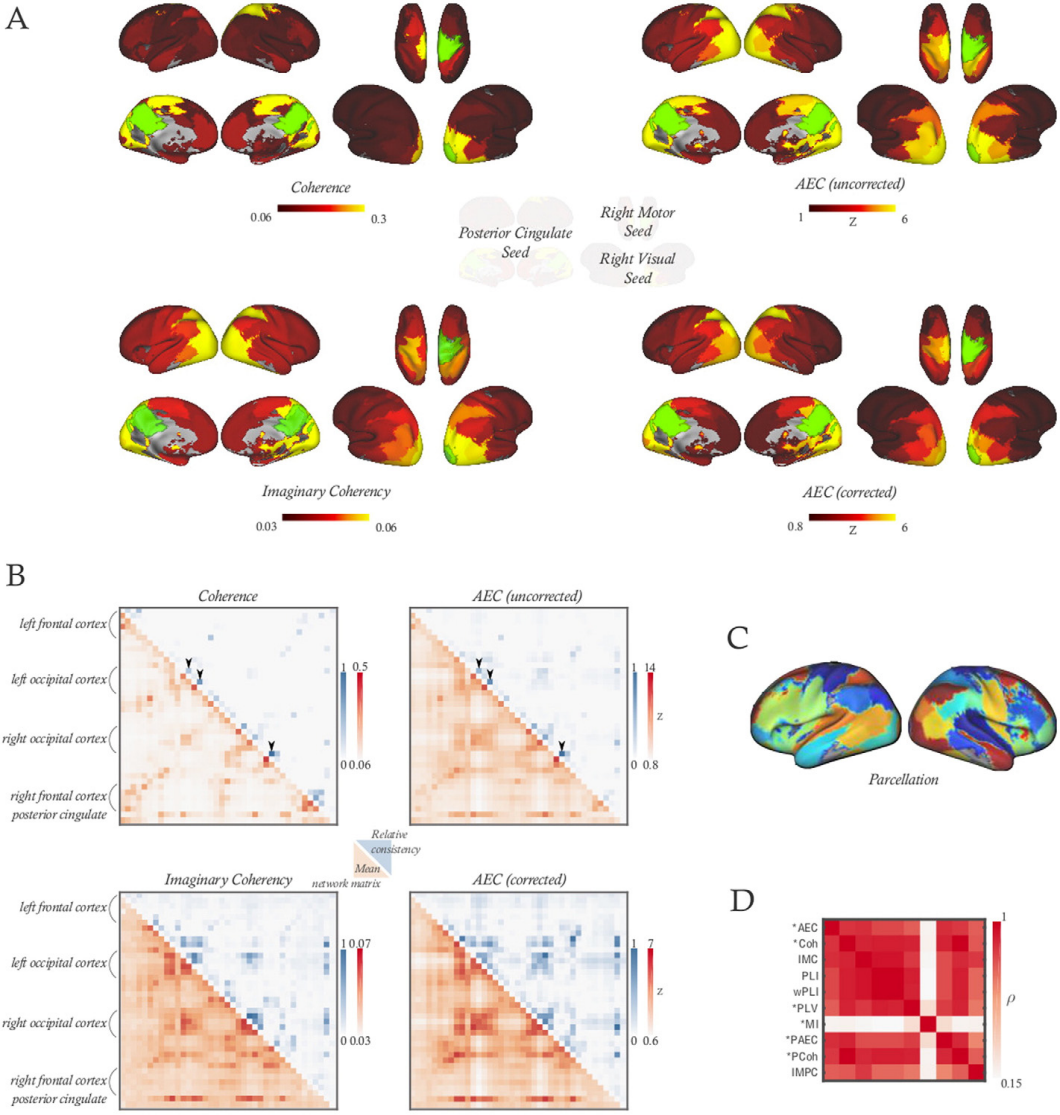
Mean alpha-band network matrices at group level. (A) Views of single rows from the network matrices in B. Left, connection strength to the posterior cingulate cortex (seed ROI number 38, shown in green). Upper right, connection strengths to the right motor cortex (seed ROI number 24). Lower right, connection strengths to right occipital cortex (seed ROI number 24). (B) Mean network matrices, and the relative edge-level consistency, for a subset of four of the metrics under test: the absolute value of coherency between ROIs, the imaginary part of coherency, and the amplitude envelope correlation (AEC), both with and without a multivariate correction for source leakage. Lower triangles, in red, show the mean network matrix over all 183 sessions in the dataset. The colourbar to the side indicates the scale. Correlations have been converted to *Z*-values before averaging. The upper triangles, in blue, indicate the relative contribution of each edge to the overall consistency of group-level network estimation, computed with Eq. (13) (c.f. Fig. 2A). The two network matrices which are susceptible to source leakage (AEC and Coherence, top row) are dominated, even at group level, by a few strong edges (highlighted with arrows), and these drive the high consistency of network estimation. (C) The parcellation of 39 fMRI-derived ROIs used for this connectivity analysis (reproduced with permission from Colclough et al. (2015)). (D) Similarity between group-level network matrices. The heat map shows the high correlations between network matrices inferred at the group-level using the undirected network measures that are either immune to source leakage bias, or have been corrected for source leakage bias (starred). AEC - amplitude envelope correlation; PAEC - partial amplitude envelope partial correlation; PLI - phase lag index; wPLI - weighted phase lag index; PLV - phase locking value; MI - mutual information of phases. Coh - band-averaged coherence; IMC - band-averaged imaginary component of coherency; PCoh - partial coherence; IMPC - partial imaginary coherence.

We find that the group-level connectivity matrices derived from the parcellation of 39 ROIs all exhibit the basic structure of connectivity expected in the resting-state, with segregated sensory networks. The posterior cingulate cortex is highly connected through the parietal and occipital lobes, as would be expected for part of the default mode network (although, as in Maldjian et al. (2014), with low connectivity to the anterior cingulate). In Fig. 1A-B, we illustrate alpha-band connectivity for three of the most common network analysis methods (amplitude envelope correlation, with and without a correction for source leakage; coherence and the imaginary part of coherency). Renderings of these networks in 3D and group-level network matrices for all methods under test are presented in the supplementary material. A listing of the locations of the 39 ROIs is given in Table 2.

General features of these network matrices are very similar over all network measures of a given type (partial, marginal, directed). We assessed the extent to which these different network measures are capturing similar information by correlating the mean edge strengths at group level computed with different metrics (shown for undirected metrics immune to, or corrected for, spatial leakage in Fig. 1D). Three-quarters of these between-metric comparisons were correlated with *ρ* ≥ 0.7.

### Consistency of group inference

**Fig. 2:**
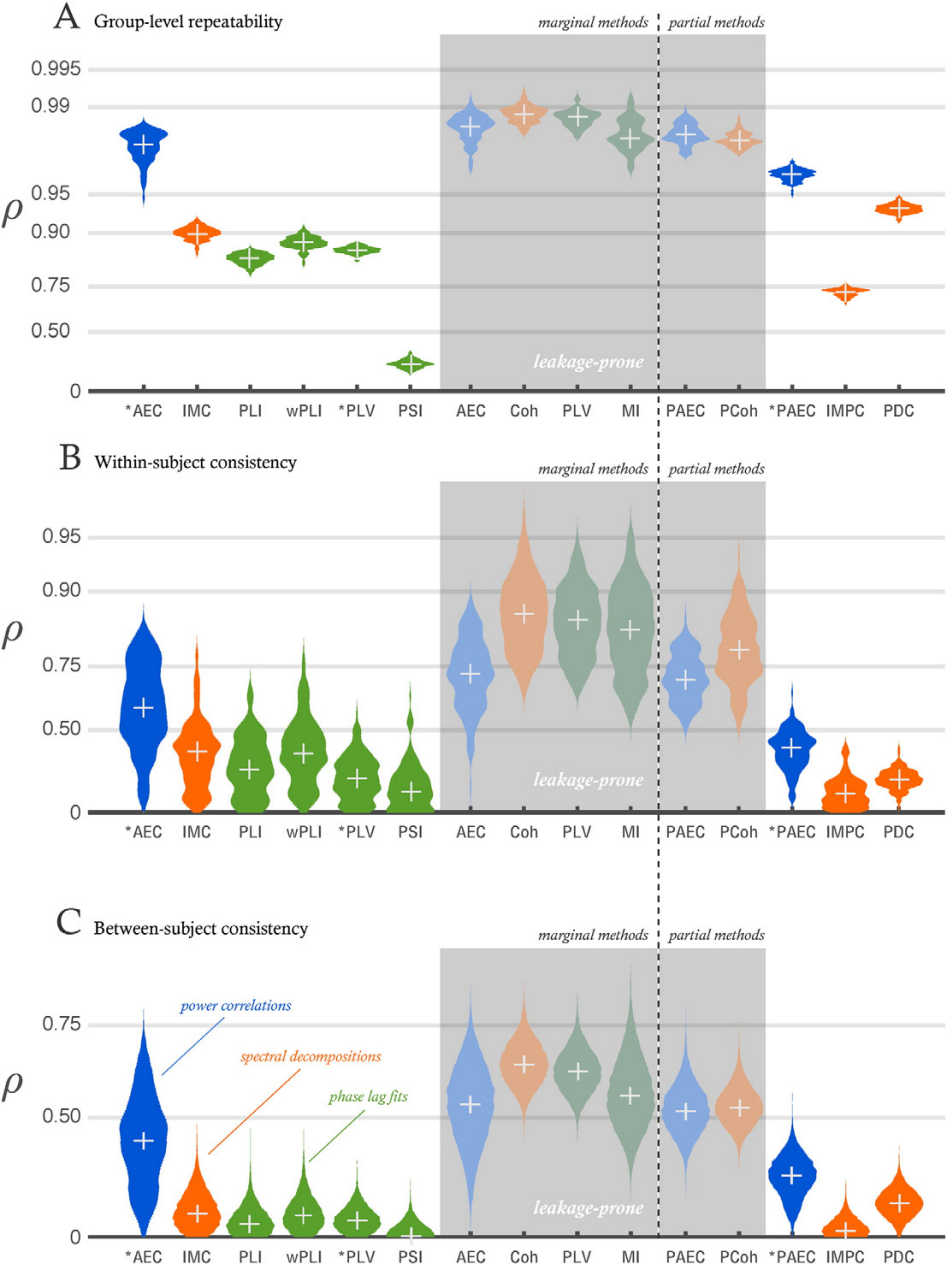
Consistency of network matrix estimation. (A) Stability of group-level inference. Correlations between network matrices inferred from separate halves of the HCP dataset, using resting-state recordings in the alpha band. The dataset was randomly partitioned in half 100 times, and the distribution of correlations between the network edge strengths in each half was produced over the bootstrapped samples. (B) Within-subject consistency of network inference. Correlations between alpha-band network matrices inferred from each of three resting-state sessions from 61 subjects of the HCP dataset. (C) Between-subject consistency of network inference. Correlations between alpha-band network matrices inferred from 61 subjects of the HCP dataset. In all cases, the violin plots show smoothed histograms of these distributions, the median marked by a white cross. Correlations have been converted to *Z*-values using Fisher's transformation, but labelled with the original rho value, for clearer display. Darker distributions with background shading identify metrics which are known to suffer from source-leakage confounds. Metrics with a star indicate that a multivariate source leakage correction has been applied before computation of the network measure. AEC - amplitude envelope correlation; PAEC - partial amplitude envelope partial correlation; PLI - phase lag index; wPLI - weighted phase lag index; PLV - phase locking value; PSI - phase slope index; MI - mutual information of phases. Coh - band-averaged coherence; IMC - band-averaged imaginary component of coherency; PCoh - partial coherence; IMPC - partial imaginary coherence; PDC - partial directed coherence.

We investigated the consistency of group-level inference by repeated boot-strapped division of the entire dataset into two equal halves, and comparing the networks inferred from each half. Fig. 2A gives the distributions of correlations between each of the two dataset halves (about 90 sessions in each), for all metrics studied here, in the alpha band. Those metrics that are affected by spatial leakage effects—amplitude correlation and partial correlation, phase locking value, coherence, mutual information—show strong consistency in group level estimation (with correlations between the two halves around 0.99). Of those metrics designed to be immune to spatial leakage effects, most achieve between-group correlations of *ρ* = 0.9, but solely in the alpha band; PLI, wPLI, IMC and PSI fare poorly (*ρ* < 0.9) in all other bands (see figures in supporting information). Crucially, applying an orthogonalisation correction to remove the impact of spatial leakage effects reduces group consistency for all methods, but uniquely the correlations and partial correlations of corrected power envelopes, retain *ρ* > 0.95. Among the directed measures, PDC performs the best (median *ρ* = 0.94).

Among those metrics which are affected by spatial leakage, the same small subset of network edges dominated the high levels of correlation between the split halves. Fig. 1B shows as heat maps the edge-wise contribution to the correlations between network matrices inferred from each half of the data, for networks inferred using coherence, imaginary coherency, correlation and leakage-corrected correlation. When spatial leakage between regions remains uncorrected (top half of Fig. 1B), a small subset of edges close to the diagonal show a very strong contribution to the consistency of group network matrix estimation, and this subset becomes much less important once the leakage-induced connections are accounted for.

### Reliability of single-subject inference

The extent to which repeated measurements of resting-state activity within the same subject can produce similar network matrices can be a good test of the reliability of a network measure. We compare correlations of network edge strengths between three consecutive resting-state recordings of each subject, for each of our tested network measures, in Fig. 2B. Again, we find high levels of repeatability among those metrics affected by spatial leakage (median correlations for AEC, partial correlation, PLV, Coherence and MI lie between 0.7 and 0.9). This repeatability is destroyed by the orthogonalisation procedure for MI, Coherence and PLV. Network measures designed to be immune to zero-lag correlation effects show large amounts of variability over subjects in the extent to which repeated sessions can reproduce the same network structure: correlations vary between 0 and 0.7, with the medians for PLI, wPLI, and the imaginary part of coherency lying around *ρ* = 0.3. Of the measures immune to, or corrected for, spatial leakage, AEC alone, and to some extent, partial correlation, when applied to leakage-corrected data, retain moderate amounts of between session repeatability; although the distributions are broad (the central 95% of within-subject network edge correlations for amplitude correlations between leakage-corrected power envelopes is 0.1–0.8).

### Consistency of network matrices between subjects

Lastly, the consistency of network matrices inferred from different subjects speaks both to the amount of between-subject variability present in healthy datasets, and to the extent to which the population average of a network measure is reflected in single-subject measurements. In our assessment of the HCP dataset, we find that network metrics sensitive to artificial correlations induced by spatial leakage retain high consistency between subjects. Of those which ignore zero-lag effects, or are corrected for leakage by orthogonalisation, AEC (median *ρ* = 0.4), with the noisier partial correlation measure (median *ρ* = 0.3) a not-too-close second, show the largest amount of between-subject correlation.

## Discussion

We have assessed a range of network measures commonly used to evaluate connectivity in electrophysiological recordings. We were interested in understanding which network measures, when applied to resting-state recordings, are able (i) to produce repeatable inference at group level, and which show consistent patterns of connectivity both (ii) between different healthy subjects, and (iii) on repeated measures of the same subject.

We find that, at a very broad level, the patterning of connectivity described by different network measures is very similar. While this is consistent with the idea that multiple methods of cortical communication are simultaneously employed, as reported for shorter-range connections in Mehrkanoon et al. (2014), these network measures may simply be picking up different characteristics of the information transfer in the data. Alternatively, the different measures may not be independent estimators of different coupling mechanisms—the dependencies between phase-based metrics and cross-correlation have been discussed by Aydore et al. (2013). However, it is also clear that we frequently find strong connections, in all or most network metrics, between ROIs with strong (high power) signals. Thus, we find dense connections both to and within visual cortex for the alpha band, and strong motor connectivity in beta (Fig. 1A). None of these observations are unexpected, but a signal strength bias should be recognised as a potential source of similarity between the network models under test here.

The confounds introduced into network estimation due to the spatial leakage of inferred cortical sources, a symptom of the broadness of the M/EEG point spread function, appear highly repeatable over scans and between subjects. We observe the highest levels of repeatability of group-level inference, and the highest consistency of network matrix estimation from single sessions, for those metrics known to be affected by zero-lag phase coupling. Versions of these metrics which regress out the shared signal (leakage-corrected envelope correlation and partial correlation), or ignore coupling with zero phase difference (PLI, PSI, imaginary part of coherency), all exhibit a large relative drop in consistency. The influence of spatial leakage is profound; clearly visible in the inferred group network matrices, when corrected and uncorrected metrics are compared (top of Fig. 1B: compare power envelope correlation with and without orthogonalisation, or coherence and the imaginary part of coherency, for example); and generating sufficiently many consistent, strong, spurious network connections over repeated measurement sessions and with different subjects to artificially inflate measures of repeatability between inferred networks. We do not, therefore, recommend the use of any network measure which cannot obviate the spurious connections engendered by spatial leakage effects.

Not all zero-lag connectivity is necessarily spurious. There is a body of experimental and theoretical work that describes the presence and potential mechanisms for zero-lag synchronisation of neural oscillations (Gollo et al., 2014, Roelfsema et al., 1997). Methods for signal reconstruction that can suppress contributions from other cortical sources (Dalal et al., 2006, Hui et al., 2010, Hauk and Stenroos, 2014) may allow the detection of these effects. However, using standard beamformers or minimum-norm source estimates, it is not possible to disambiguate spatial leakage effects from zero-lag synchrony of biological origin (although see Wens et al. (2015) for recent work constructing models of field spread for pair-wise connectivity estimation). Because the impact of the spurious connections is considerable (see above), we (and others, Schoffelen and Gross (2009), Palva and Palva (2012), Hipp et al. (2012)) argue that interpretable connectivity estimation is only possible when zero-lag connections are removed or otherwise ignored.

The most consistent network measure that is corrected for the biases induced by spatial leakage artefacts, and which we can recommend for use, is the correlation between band-limited power envelopes, after an orthogonalisation correction to remove shared zero-lag signals. It is considered good practice (Smith et al., 2013) to use network measures which elucidate only direct connections, if the resulting network matrices are desired to be physiologically interpretable, rather than simply used for discriminative purposes. These partial methods tend to be noisier to estimate, even with regularisation to encode underlying assumptions of sparsity in the connectivity pattern. (Although in high-quality fMRI recordings, partial methods are found to be more discriminative than marginal approaches (Smith et al., 2011, Smith et al., 2013).) Of these, we believe that the partial correlations between leakage-corrected power envelopes show sufficient repeatability, particularly in group-level inference, to be recommended for most resting state experiments. We should recognise that these recommendations are for methods that rely on orthogonalisation corrections for their removal of source leakage. This correction is optimal only for Gaussian-distributed data (Brookes et al., 2014), and if this assumption badly fails, these methods may have inflated performance for the reasons discussed above. If directional connectivity estimation is required, a large amount of repeatability is sacrificed, but the PDC performed best out of the directed metrics on test. (Some concerns also exist about the robustness of PDC to leakage artefacts, see comment above in Methods Section 2.5.3.)

We find, over all frequency bands, phase-based measures and imaginary partial coherence to perform the worst on all assessments, in particular showing very poor within-subject repeatibility. Why do these measures exhibit poor performance? We reject the notion that they do not measure anything related to cortical connectivity: there is a wide literature of interpretable results using these measures, and in our data they produce sensible connectivity structure at group level, and show better within-subject consistency than between-subject. Their acceptably-high repeatability for group-level inference, and well-known performance with large task datasets, suggest that these metrics may simply be noisier estimators than the others on test, leading to a reduced ability to extract a consistent basis of functional connectivity from noisy recordings. Longer resting-state scans than are used here, perhaps of fifteen minutes or more, may be required to sufficiently increase the SNR that these measures improve performance at the single-subject level. We note also that Hardmeier et al. (2014) find good test-retest reliability with PLI for sensor-level EEG analyses (intra-class correlation coefficients of 0.6–0.8) using recordings of 12 min. We have restricted ourselves to a study of repeatability, and so it may be that (despite the strong correlations between group-level results between each metric) some phase-based methods are more sensitive than others to network covariation with subject attributes or transient connectivity features. Further, we have only performed our analysis using a data-driven parcellation from fMRI, and it may be that some metrics would show improved performance should a reliable MEG parcellation driven by the patterns of phase-based connectivity within the data become available. Lastly, it is possible that the beamformer used for source reconstruction shows variability between runs, for example in estimating the source orientations. While this would affect all of our tests equally, it is a concern which is yet to be fully explored in the literature.

In this study, we have focussed on the behaviour of connectivity measures commonly employed in MEG and EEG, applied to a large resting-state dataset. We believe that most of our conclusions should carry over to many task paradigms, where cortical responses can be more repeatable between trials and subjects than in the resting state. However, it may be that this inherent repeatability in response boosts the SNR of many connectivity metrics when connections are averaged over trials, such that poorly-performing measures in our study, such as the PLI, become efficacious.

It is becoming established practice that resting-state connectivity analyses in MEG and EEG are carried out in source space, using a set of functionally-defined regions which cover as much of the cortex as possible given the recorded data. We further suggest employing network measures that are inured to the connectivity confounds introduced by source leakage profiles. To satisfy all these purposes, our results suggest that the most consistent connectivity measure to employ for resting-state studies is the correlation between orthogonalised, band-limited, power envelopes.

## Figures and Tables

**Table 1:** Classification of the network metrics tested. We separate the connectivity estimation methods by the inference process; whether or not only direct network edges are found (partial methods) or direct and indirect connections (marginal methods); whether or not directionality is ascribed to each edge; and whether or not the method is robust against spatial leakage artefacts. ^★^PDC may be sensitive to magnetic field spread, see discussion in Methods Section 2.5.3.

Abbreviation	Connectivity metric	Type	Direct associations	Causal relations	Leakage-corrected
AEC	Amplitude envelope correlation	Amplitude coupling	Marginal	Undirected	Yes, with orthogonalisation
PAEC	Amplitude envelope partial correlation	Amplitude coupling	Partial	Undirected	Yes, with orthogonalisation
Coh	Absolute coherence	Spectral coherence	Marginal	Undirected	No
IMC	Imaginary coherency	Spectral coherence	Marginal	Undirected	Yes
PCoh	Partial coherence	Spectral coherence	Partial	Undirected	No
IMPC	Imaginary partial coherency	Spectral coherence	Partial	Undirected	Yes
PLV	Phase-locking value	Phase estimation	Marginal	Undirected	No
PLI	Phase lag index	Phase estimation	Marginal	Undirected	Yes
wPLI	Weighted phase lag index	Phase estimation	Marginal	Undirected	Yes
PSI	Phase slope index	Phase estimation	Marginal	Directed	Yes
MI	Mutual information between phases	Phase estimation	Marginal	Undirected	No
PDC	Partial directed coherence	Auto-regressive modelling	Partial	Directed	Yes^★^

**Table 2:** Index of ROI numbers.

ROI number	ROI location
1, 2, 3, 4, 5	Left frontal lobe
6	Left somatosensory cortex
7, 8	Left motor cortex
9, 10, 11	Left parietal cortex
12, 13	Left visual cortex
14, 15	Left occipital lobe
16, 17, 18	Left temporal lobe
19, 20, 21	Right temporal lobe
22, 23	Right occipital lobe
24, 25	Right visual cortex
26, 27, 28, 29, 30	Right parietal lobe
31	Right motor cortex
32	Right somatosensory cortex
33, 34, 35, 36, 37	Right frontal lobe
38	Posterior cingulate cortex
39	Medial frontal cortex

## Supplementary data

Supplementary figures Image 1

AEC uncorrected group network matrix, alpha band. Image 2

AEC corrected group network matrix, alpha band. Image 3

Coherence group network matrix, alpha band. Image 4

IMC group network matrix, alpha band. Image 5

## Footnotes

1 Unavailable subjects by ID: 104012, 125525, 151526, 182840, 200109, 500222.

2 http://pub.ist.ac.at/~schloegl/matlab/tsa/

## References

[bb0005] Astolfi L., Cincotti F., Mattia D., Marciani M.G., Salinari S., de Vico Fallani F., Baccala L.A., Ursino M., Zavaglia M., Ding L., Edgar J.C., Miller G.A., He B., Babiloni F. (2007). Comparison of different cortical connectivity estimators for high-resolution EEG recordings. Hum. Brain Mapp..

[bb0010] Aydore S., Pantazis D., Leahy R.M. (2013). A note on the phase locking value and its properties. NeuroImage.

[bb0015] Baccala L.A., Sameshima K. (2001). Partial directed coherence: a new concept in neural structure determination. Biol. Cybern..

[bb0020] Baker A.P., Brookes M.J., Rezek A., Smith S.M., Behrens T.E., Smith P.J.P., Woolrich M.W. (2014). Fast transient networks in spontaneous human brain activity. eLIFE.

[bb0025] Brookes M.J., Stevenson C.M., Barnes G.R., Hillebrand A., Simpson M.I.G., Francis S.T., Morris P.G. (2007). Beamformer reconstruction of correlated sources using a modified source model. NeuroImage.

[bb0030] Brookes M.J., Woolrich M.W., Luckhoo H., Price D., Hale J.R., Stephenson M.C., Barnes G.R., Smith S.M., Morris P.G. (2011). Investigating the electrophysiological basis of resting state networks using magnetoencephalography. Proc. Natl. Acad. Sci. U. S. A..

[bb0035] Brookes M.J., Woolrich M.W., Barnes G.R. (2012). Measuring functional connectivity in MEG: a multivariate approach insensitive to linear source leakage. NeuroImage.

[bb0040] Brookes M.J., O'Neill G.C., Hall E.L., Woolrich M.W., Baker A., Palazzo-Corner S., Robson S.E., Barnes G.R. (2014). Measuring temporal, spectral and spatial changes in electrophysiological brain network connectivity. NeuroImage.

[bb0045] Colclough G.L., Brookes M.J., Smith S.M., Woolrich M.W. (2015). A symmetric multivariate leakage correction for MEG connectomes. NeuroImage.

[bb0050] Dalal S.S., Sekihara K., Nagarajan S.S. (2006). Modified beamformers for coherent source region suppresion. IEEE Trans. Biomed. Eng..

[bb0055] de Pasquale F., Della Penna S., Snyder A.Z., Lewis P.S., Mantini D., Marzetti L., Belardinelli P., Ciancetta L., Pizzella V., Romani G.L., Corbetta M. (2010). Temporal dynamics of spontaneous MEG activity in brain networks. Proc. Natl. Acad. Sci. U. S. A..

[bb0060] de Pasquale F., Della Penna S., Sporns O., Romani G.L., Corbetta M. (2015). A dynamic core network and global efficiency in the resting human brain. Cereb. Cortex.

[bb0065] Deuker L., Bullmore E.T., Smith M., Christensen S., Nathan P.J., Rockstroh B., Bassett D.S. (2009). Reproducability of graph metrics of human brain functional networks. NeuroImage.

[bb0070] Gollo L.L., Mirasso C., Sporns O., Breakspear M. (2014). Mechanisms of zero-lag synchronization in cortical motifs. PLoS Comput. Biol..

[bb0075] Granger C.W.J. (1969). Investigating causal relations by econometric models and cross-spectral methods. Econometrica.

[bb0080] Hardmeier M., Hatz F., Bousleiman H., Schindler C., Stam C.J., Fuhr P. (2014). Reproducability of functional connectivity and graph measures based on the phase lag index (PLI) and weighted phase lag index (WPLI) derived from high resolution EEG. PLoS ONE.

[bb0085] Hauk O., Stenroos M. (2014). A framework for the design of flexible cross-talk functions for spatial filtering of eeg/meg data: DeFleCT. Hum. Brain Mapp..

[bb0090] Hipp J.F., Hawellek D.J., Corbetta M., Siegel M., Engel A.K. (2012). Large-scale cortical correlation structure of spontaneous oscillatory activity. Nat. Neurosci..

[bb0095] Hui H.B., Pantazis D., Bressler S.L., Leahy R.M. (2010). Identifying true cortical interactions in meg using the nulling beamformer. NeuroImage.

[bb0100] Jin S.H., Seol J., Kim J.S., Chung C.K. (2011). How reliable are the functional connectivity networks of MEG in resting states?. J. Neurophys..

[bb0105] Kaminski M., Blinowska K.J. (2014). Directed transfer function is not influenced by volume conduction—inexpedient pre-processing should be avoided. Front. Comput. Neurosci..

[bb0110] Lachaux J.P., Rodriguez E., Martinerie J., Varela F.J. (1999). Measuring phase synchrony in brain signals. Hum. Brain Mapp..

[bb0115] Lachaux J.P., Rodriguez E., le van Quyen M., Lutz A., Martinerie J., Varela F.J. (2000). Studying single-trials of phase synchronous activity in the brain. Int. J. Bifurcation Chaos.

[bb0120] Larson-Prior L.J., Oostenveld R., Della Penna S., Michalareas G., Prior F., Babajani-Feremi A., Schoffelen J.M., Marzetti L., de Pasquale F., Di Pompeo F., Stout J., Woolrich M.W., Luo Q., Bucholz R., Fries P., Pizella V., Romani G.L., Corbetta M., Snyder A.Z., Consortium, W.M.H. (2013). Adding dynamics to the human connectome project with MEG. NeuroImage.

[bb0125] Leistritz L., Pester B., Doering A., Schiecke K., Babiloni F., Astolfi L., Witte H. (2013). Time-variant partial directed coherence for analysing connectivity: a methodological study. Phil. Trans. R. Soc. A.

[bb0130] Luckhoo H., Hale J.R., Stokes M.G., Nobre A.G., Morris P.G., Brookes M.J., Woolrich M.W. (2012). Inferring task-related networks using independent component analysis in magnetoencephalography. NeuroImage.

[bb0135] Maldjian J.A., Davenport E.M., Whitlow C.T. (2014). Graph theoretical analysis of resting-state MEG data: identifying interhemispheric connectivity and the default mode. NeuroImage.

[bb0140] Marrelec G., Krainik A., Duffau H., Pelegrini-Issac M., Lehericy S., Doyon J., Benali H. (2006). Partial correlation for functional brain interactivity investigation in functional MRI. NeuroImage.

[bb0145] Marzetti L., Della Penna S., Snyder A.Z., Pizzella V., Nolte G., de Pasquale F., Romani G.L., Corbetta M. (2013). Frequency specific interactions of MEG resting state activity within and across brain networks as revealed by the multivariate interaction measure. NeuroImage.

[bb0150] Mazumder R., Hastie T. (2012). The graphical lasso: new insights and alternatives. Electron. J. Stat..

[bb0155] Mehrkanoon S., Breakspear M., Britz J., Boonstra T.W. (2014). Intrinsic coupling modes in source-reconstructed electroencephalography. Brain Connectivity.

[bb0160] Nolte G., Bai O., Wheaton L., Mari Z., Vorbach S., Hallett M. (2004). Identifying true brain interaction from EEG using the imaginary part of coherency. J. Clin. Neurophysiol..

[bb0165] Nolte G., Ziehe A., Nikulin V.V., Schlögl A., Krämer N., Brismar T., Müller K.R. (2008). Robustly estimating the flow direction of information in complex physical systems. Phys. Rev. Lett..

[bb0170] O'Neill G.C., Bauer M., Woolrich M.W., Morris P.G., Barnes G.R., Brookes M.J. (2015). Dynamic recruitment of resting state sub-networks. NeuroImage.

[bb0175] Omidvarnia A., Azemi G., Boashash B., O’Toole J.M., Colditz P., Vanhatalo S. (2014). Measuring time-varying information flow for scalp EEG signals: orthogonalized partial directed coherence. IEEE Trans. Biomed. Eng..

[bb0180] Paluš M. (1997). Detecting phase synchronization in noisy systems. Phys. Lett. A.

[bb0185] Palva S., Palva J.M. (2012). Discovering oscillatory interaction networks with M/EEG: challenges and breakthroughs. Trends Cogn. Sci..

[bb0190] Peng H., Long F., Ding C. (2005). Feature selection based on mutual information: criteria of max-dependency, max-relevance and min-redundancy. IEEE Trans. Pattern Anal. Mach. Intell..

[bb0195] Robinson S.E., Vrba J., Yoshimoto T., Kotani M., Kuriki S., Karibe H., Nakasato N. (1999). Functional neuroimaging by Synthetic Aperture Magnetometry (SAM). Recent Advances in Biomagnetism.

[bb0200] Roelfsema P.R., Engel A.K., König P., Singer W. (1997). Visuomotor integration is associated with zero time-lag synchronization among cortical areas. Nature.

[bb0205] Schoffelen J.M., Gross J. (2009). Source connectivity analysis with MEG and EEG. Hum. Brain Mapp..

[bb0210] Smith S.M., Beckmann C.F., Ramnani N., Woolrich M.W., Bannister P.R., Jenkinson M., Matthews P.M., McGonigle D.J. (2005). Variability in fMRI: a re-examination of inter-session differences. Hum. Brain Mapp..

[bb0215] Smith S.M., Miller K.L., Salimi-Khorshidi G., Webster M., Beckmann C.F., Nichols T.E., Ramsey J.D., Woolrich M.W. (2011). Network modelling methods for FMRI. NeuroImage.

[bb0220] Smith S.M., Vidaurre D., Beckmann C.F., Glasser M.F., Jenkinson M., Miller K.L., Nichols T.E., Robinson E., Salimi-Khorshidi G., Woolrich M.W., Barch D.M., Ugurbil K., Van Essen D.C. (2013). Functional connectomics from resting-state fMRI. Trends Cogn. Sci..

[bb0225] Stam C.J. (2014). Modern network science of neurological disorders. Nat. Rev. Neurosci..

[bb0230] Stam C.J., van Straaten E.C.W. (2012). The organization of physiological brain networks. Clin. Neurophysiol..

[bb0235] Stam C.J., Nolte G., Daffertshofer A. (2007). Phase lag index: assessment of functional connectivity from multi channel EEG and MEG with diminished bias from common sources. Hum. Brain Mapp..

[bb0240] Stam C.J., de Haan W., Daffertshofer A., Jones B.F., Manshanden I., van Cappellen van Walsum A.M., Montez T., Verbunt J.P.A., de Munck J.C., van Dijk B.W., Berendse H.W., Sheltens P. (2009). Graph theoretical analysis of magnetoencephalographic functional connectivity in Alzheimer's disease. Brain.

[bb0245] Tewarie P., Hillebrand A., van Dellen E., Schoonheim M.M., Barkhof F., Polman C.H., Beauliue C., Gong G., van Dijk B.W., Stam C.J. (2014). Structural degress predicts functional network connectivity: a multimodal resting-state fMRI and MEG study. NeuroImage.

[bb0250] Van Essen D.C., Smith S.M., Barch D.M., Behrens T.E., Yacoub E., Ugurbil K., for the WU-Minn HCP Consortium (2013). The WU-Minn human Connectome project: an overview. NeuroImage.

[bb0255] van Straaten E.C.W., Stam C.J. (2013). Structure out of chaos: functional brain network analysis with EEG, MEG and functional MRI. Eur. Neuropsychopharmacol..

[bb0260] Van Veen B.D., van Drongelen W., Yuchtman M., Suzuki A. (1997). Localization of brain electrical activity via linearly constrained minimum variance spatial filtering. IEEE Trans. Biomed. Eng..

[bb0265] Vinck M., Oostenveld R., van Wingerden M., Battaglia F., Pennartz C.M.A. (2011). An improved index of phase-synchronization for electrophysiological data in the presence of volume-conduction, noise and sample bias. NeuroImage.

[bb0270] Wang H.E., Benar C.G., Quilichini P.P., Friston K.J., Jirsa V., Bernand C. (2014). A systematic framework for functional connectivity measures. Front. Neurosci..

[bb0275] Wens V., Marty B., Mary A., Op de Beeck M., Goldman S., Van Bogaert P., Peigneux P., De Tiege X. (2015). A geometric correction scheme for spatial leakage effects in MEG/EEG seed-based functional connectivity mapping. Hum. Brain Mapp..

[bb0280] Wilmer A., de Lussanet M., Lappe M. (2012). Time-delayed mutual information of the phase as a measure of functional connectivity. PLoS ONE.

[bb0285] Woolrich M.W., Hunt L., Groves A.R., Barnes G.R. (2011). MEG beamforming using Bayesian PCA for adaptive data covariance matrix regularization. NeuroImage.

