# Supplementary material for: How reliable are MEG resting-state connectivity metrics?
Source: Neuroimage. 2016 Sep;138:284–93. doi: 10.1016/j.neuroimage.2016.05.070 (PMC5056955; doi:10.1016/j.neuroimage.2016.05.070)
Supplement: Supplementary file 1 — Supplementary figures [file mmc1.pdf]

5. Supplementary Information

6. SI1: Group results in the alpha band

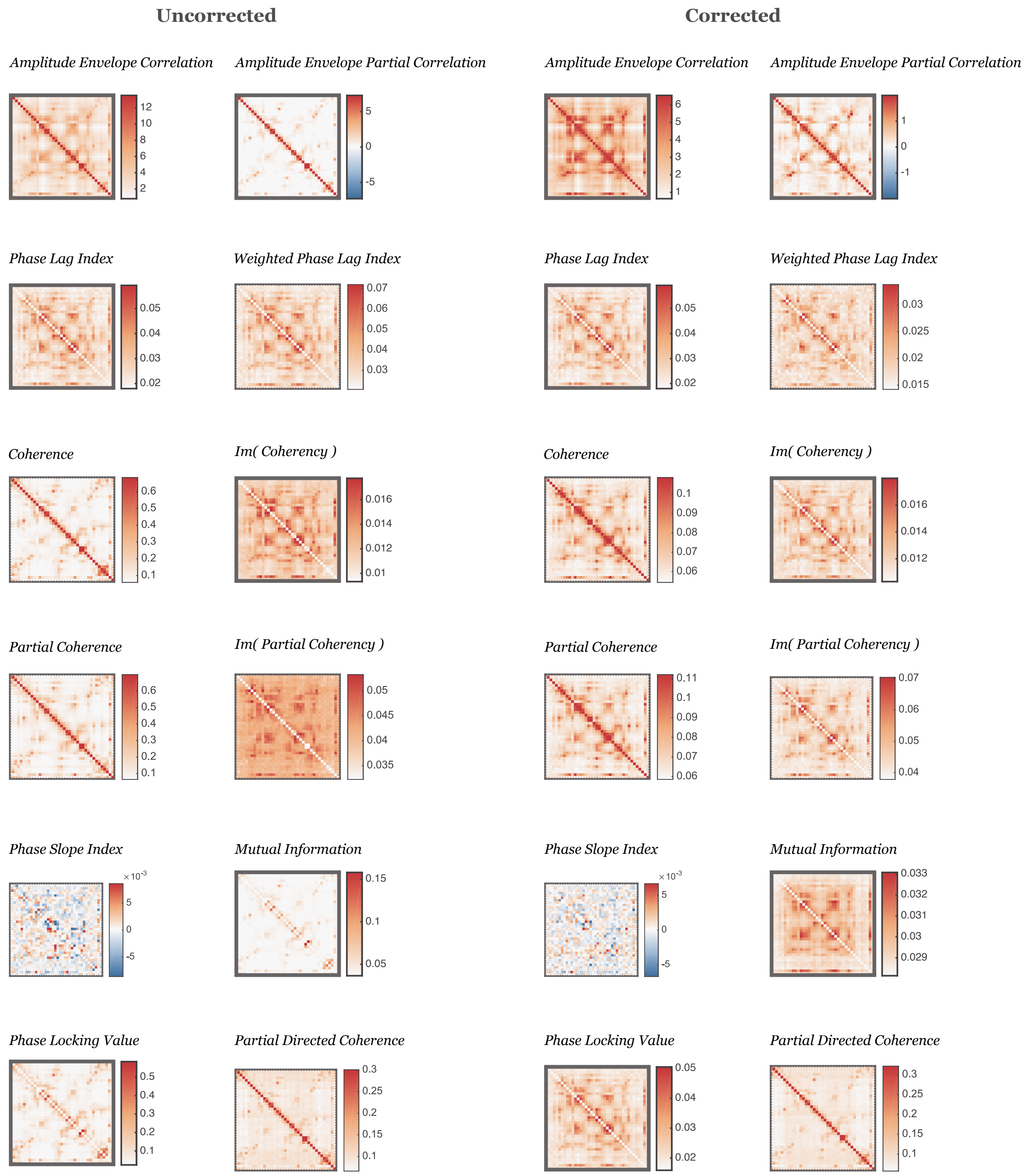

Figure 3: Mean alpha-band network matrices at the group level inferred for all 183 sessions in the HCP dataset, for all metrics under test.

7. SI3: Full results using 10-second windows for phase and spectrum estimation

7.1. Split-half reliability

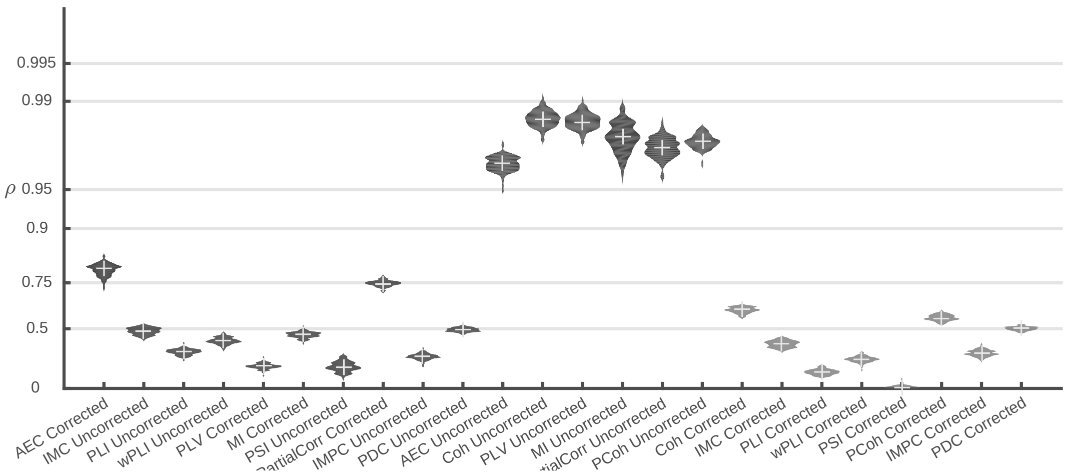

Figure 4: Stability of delta-band (1–4 Hz) group-level inference. The figure displays correlations between network matrices inferred from separate halves of the HCP dataset, using resting-state recordings in the delta band. The dataset was randomly partitioned in half 100 times, and the distribution of correlations between the network edge strengths in each half produced over the bootstrapped samples. The violin plots show smoothed histograms of these distributions, the median marked by a white cross. Network estimation methods are ordered to match the figure in the main text: methods immune to or corrected for spatial leakage; methods which suffer from spatial leakage; and finally methods which are immune, but which have had orthogonalisation applied for completeness. AEC - amplitude envelope correlation; PartialCorr - amplitude envelope partial correlation; PLI - phase lag index; wPLI - weighted phase lag index; PLV - phase locking value; Coh - band-averaged coherence; IMC - band-averaged imaginary component of coherency; PCoh - partial coherence; IMPC - imaginary partial coherence; PSI - phase slope index; MI - mutual information of phases; PDC - partial directed coherence.

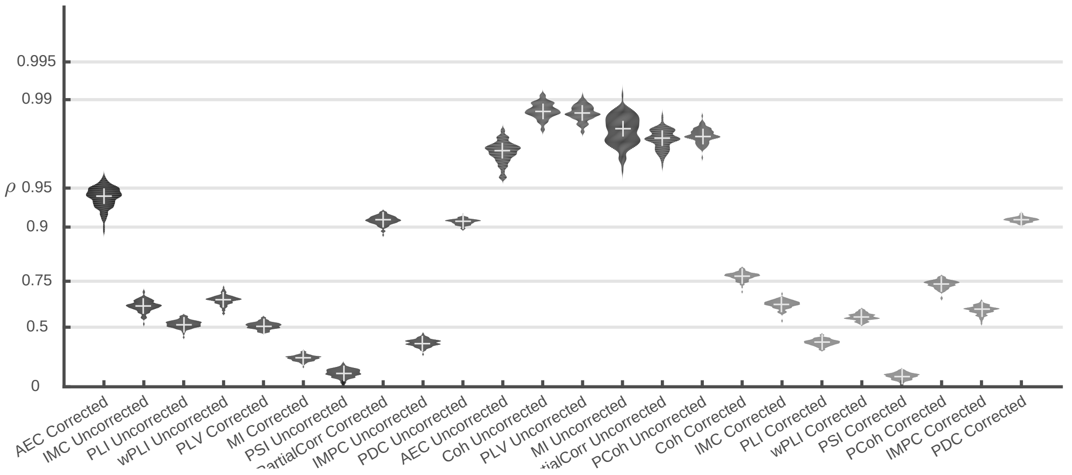

Figure 5: Stability of theta-band (4–8 Hz) group-level inference. The figure displays correlations between network matrices inferred from separate halves of the HCP dataset, using resting-state recordings in the theta band. The dataset was randomly partitioned in half 100 times, and the distribution of correlations between the network edge strengths in each half produced over the bootstrapped samples. The violin plots show smoothed histograms of these distributions, the median marked by a white cross. Network estimation methods are ordered to match the figure in the main text: methods immune to or corrected for spatial leakage; methods which suffer from spatial leakage; and finally methods which are immune, but which have had orthogonalisation applied for completeness. AEC - amplitude envelope correlation; PartialCorr - amplitude envelope partial correlation; PLI - phase lag index; wPLI - weighted phase lag index; PLV - phase locking value; Coh - band-averaged coherence; IMC - band-averaged imaginary component of coherency; PCoh - partial coherence; IMPC - imaginary partial coherence; PSI - phase slope index; MI - mutual information of phases; PDC - partial directed coherence.

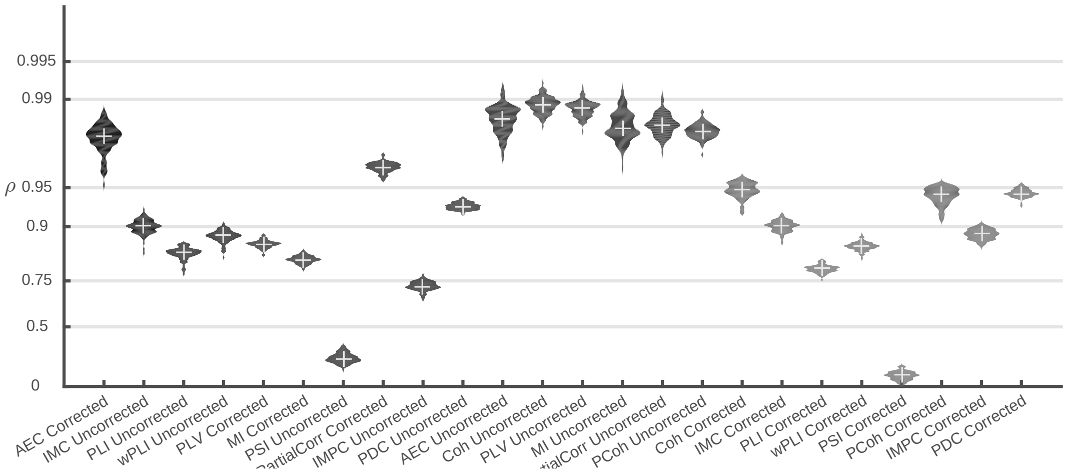

Figure 6: Stability of alpha-band (8–13 Hz) group-level inference. The figure displays correlations between network matrices inferred from separate halves of the HCP dataset, using resting-state recordings in the theta band. The dataset was randomly partitioned in half 100 times, and the distribution of correlations between the network edge strengths in each half produced over the bootstrapped samples. The violin plots show smoothed histograms of these distributions, the median marked by a white cross. Network estimation methods are ordered to match the figure in the main text: methods immune to or corrected for spatial leakage; methods which suffer from spatial leakage; and finally methods which are immune, but which have had orthogonalisation applied for completeness. AEC - amplitude envelope correlation; PartialCorr - amplitude envelope partial correlation; PLI - phase lag index; wPLI - weighted phase lag index; PLV - phase locking value; Coh - band-averaged coherence; IMC - band-averaged imaginary component of coherency; PCoh - partial coherence; IMPC - imaginary partial coherence; PSI - phase slope index; MI - mutual information of phases; PDC - partial directed coherence.

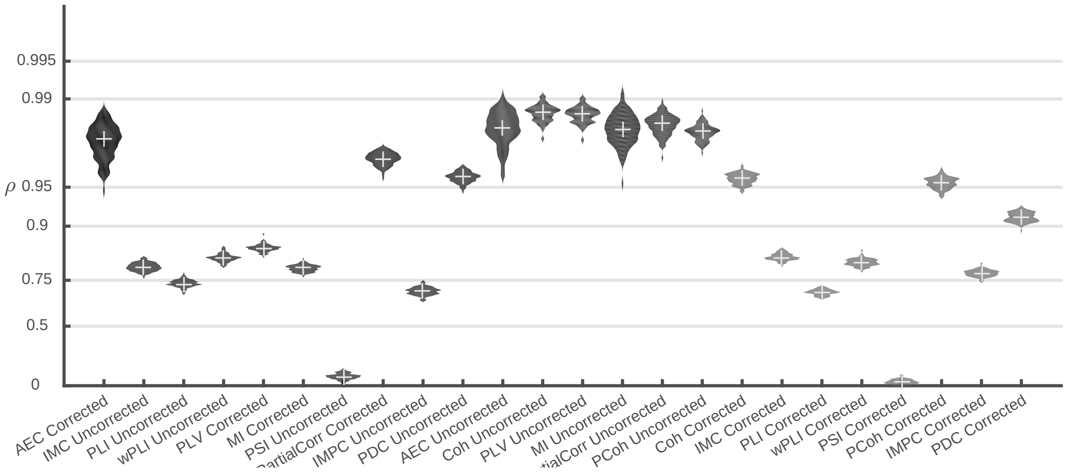

Figure 7: Stability of beta-band (13–30 Hz) group-level inference. The figure displays correlations between network matrices inferred from separate halves of the HCP dataset, using resting-state recordings in the beta band. The dataset was randomly partitioned in half 100 times, and the distribution of correlations between the network edge strengths in each half produced over the bootstrapped samples. The violin plots show smoothed histograms of these distributions, the median marked by a white cross. Network estimation methods are ordered to match the figure in the main text: methods immune to or corrected for spatial leakage; methods which suffer from spatial leakage; and finally methods which are immune, but which have had orthogonalisation applied for completeness. AEC - amplitude envelope correlation; PartialCorr - amplitude envelope partial correlation; PLI - phase lag index; wPLI - weighted phase lag index; PLV - phase locking value; Coh - band-averaged coherence; IMC - band-averaged imaginary component of coherency; PCoh - partial coherence; IMPC - imaginary partial coherence; PSI - phase slope index; MI - mutual information of phases; PDC - partial directed coherence.

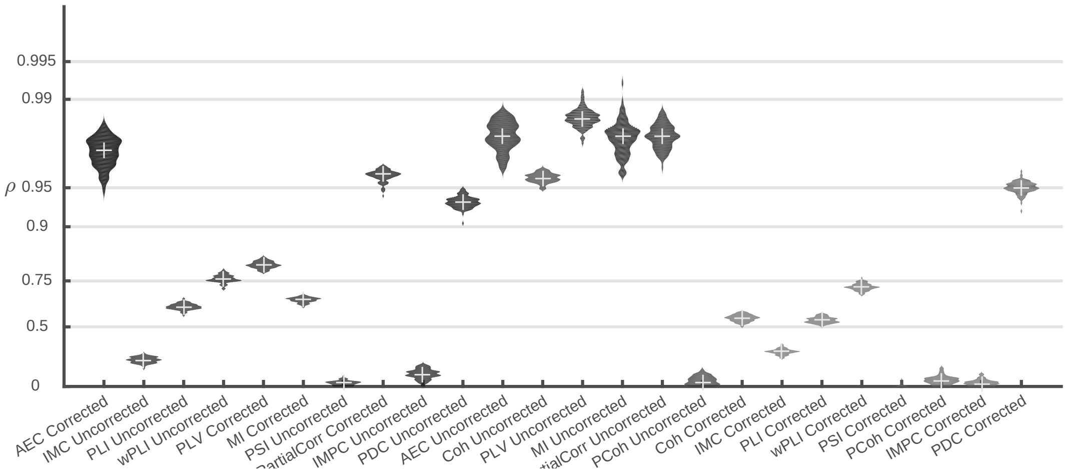

Figure 8: Stability of low-beta-band (13–20 Hz) group-level inference. The figure displays correlations between network matrices inferred from separate halves of the HCP dataset, using resting-state recordings in the beta band. The dataset was randomly partitioned in half 100 times, and the distribution of correlations between the network edge strengths in each half produced over the bootstrapped samples. The violin plots show smoothed histograms of these distributions, the median marked by a white cross. Network estimation methods are ordered to match the figure in the main text: methods immune to or corrected for spatial leakage; methods which suffer from spatial leakage; and finally methods which are immune, but which have had orthogonalisation applied for completeness. AEC - amplitude envelope correlation; PartialCorr - amplitude envelope partial correlation; PLI - phase lag index; wPLI - weighted phase lag index; PLV - phase locking value; Coh - band-averaged coherence; IMC - band-averaged imaginary component of coherency; PCoh - partial coherence; IMPC - imaginary partial coherence; PSI - phase slope index; MI - mutual information of phases; PDC - partial directed coherence.

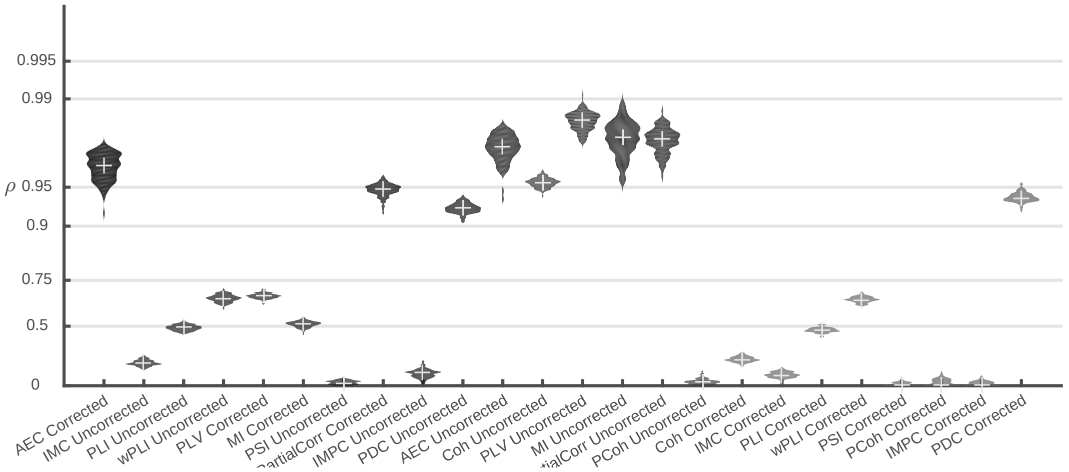

Figure 9: Stability of high-beta-band (20–30 Hz) group-level inference. The figure displays correlations between network matrices inferred from separate halves of the HCP dataset, using resting-state recordings in the beta band. The dataset was randomly partitioned in half 100 times, and the distribution of correlations between the network edge strengths in each half produced over the bootstrapped samples. The violin plots show smoothed histograms of these distributions, the median marked by a white cross. Network estimation methods are ordered to match the figure in the main text: methods immune to or corrected for spatial leakage; methods which suffer from spatial leakage; and finally methods which are immune, but which have had orthogonalisation applied for completeness. AEC - amplitude envelope correlation; PartialCorr - amplitude envelope partial correlation; PLI - phase lag index; wPLI - weighted phase lag index; PLV - phase locking value; Coh - band-averaged coherence; IMC - band-averaged imaginary component of coherency; PCoh - partial coherence; IMPC - imaginary partial coherence; PSI - phase slope index; MI - mutual information of phases; PDC - partial directed coherence.

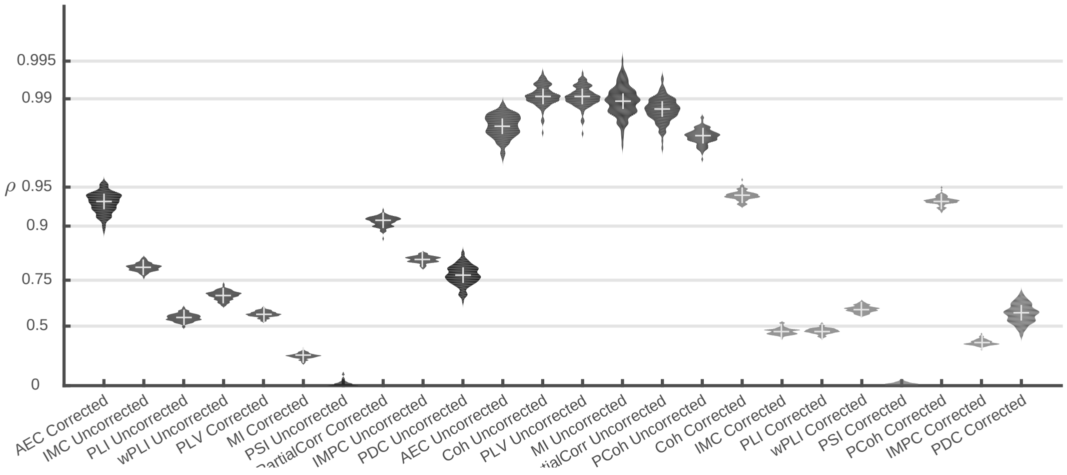

Figure 10: Stability of gamma-band (30–48 Hz) group-level inference. The figure displays correlations between network matrices inferred from separate halves of the HCP dataset, using resting-state recordings in the beta band. The dataset was randomly partitioned in half 100 times, and the distribution of correlations between the network edge strengths in each half produced over the bootstrapped samples. The violin plots show smoothed histograms of these distributions, the median marked by a white cross. Network estimation methods are ordered to match the figure in the main text: methods immune to or corrected for spatial leakage; methods which suffer from spatial leakage; and finally methods which are immune, but which have had orthogonalisation applied for completeness. AEC - amplitude envelope correlation; PartialCorr - amplitude envelope partial correlation; PLI - phase lag index; wPLI - weighted phase lag index; PLV - phase locking value; Coh - band-averaged coherence; IMC - band-averaged imaginary component of coherency; PCoh - partial coherence; IMPC - imaginary partial coherence; PSI - phase slope index; MI - mutual information of phases; PDC - partial directed coherence.

7.2. Intra-subject consistency

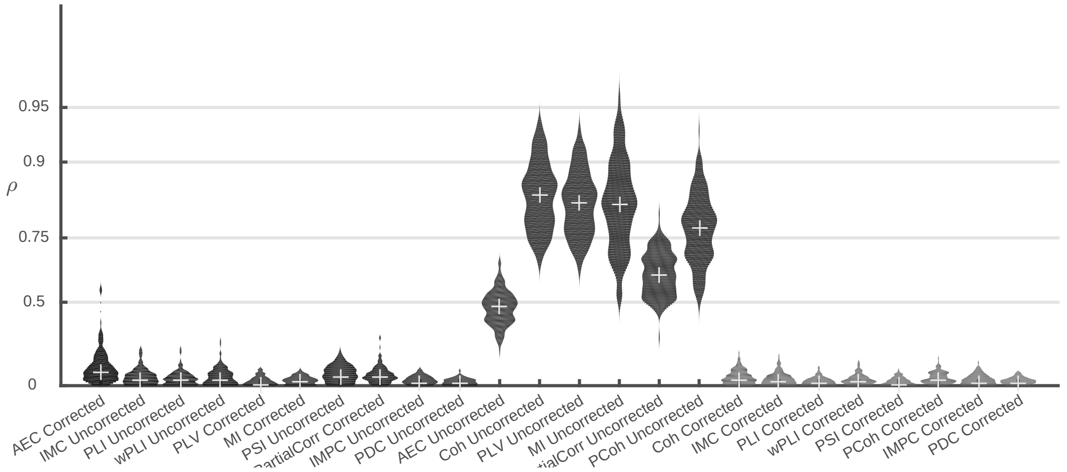

Figure 11: Within-subject consistency of delta-band (1–4 Hz) network inference. The figure shows correlations between delta-band network matrices inferred from each of three resting-state sessions from 61 subjects of the HCP dataset. The violin plots show smoothed histograms of the correlations within each subject, the median marked by a white cross. Network estimation methods are ordered to match the figure in the main text: methods immune to or corrected for spatial leakage; methods which suffer from spatial leakage; and finally methods which are immune, but which have had orthogonalisation applied for completeness. AEC - amplitude envelope correlation; PartialCorr - amplitude envelope partial correlation; PLI - phase lag index; wPLI - weighted phase lag index; PLV - phase locking value; Coh - band-averaged coherence; IMC - band-averaged imaginary component of coherency; PCoh - partial coherence; IMPC - imaginary partial coherence; PSI - phase slope index; MI - mutual information of phases; PDC - partial directed coherence.

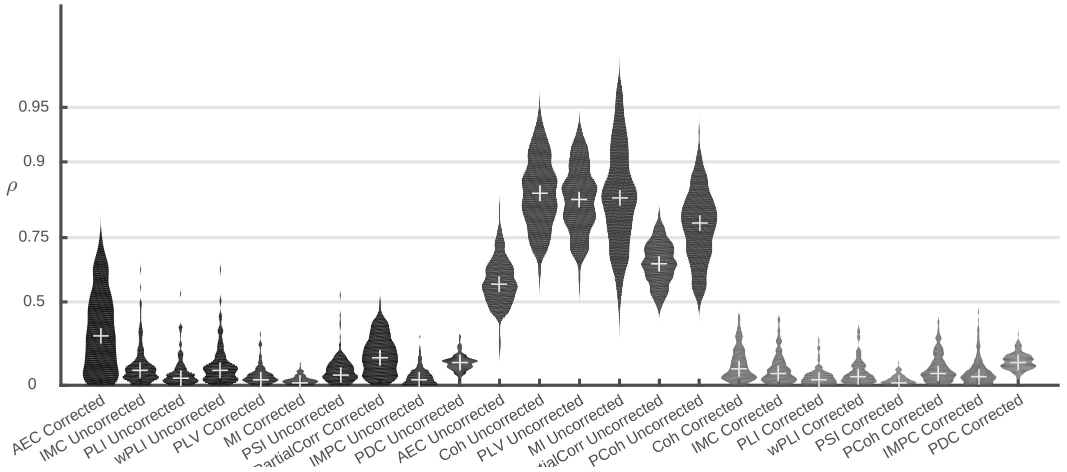

Figure 12: Within-subject consistency of theta-band (4–8 Hz) network inference. The figure shows correlations between theta-band network matrices inferred from each of three resting-state sessions from 61 subjects of the HCP dataset. The violin plots show smoothed histograms of the correlations within each subject, the median marked by a white cross. Network estimation methods are ordered to match the figure in the main text: methods immune to or corrected for spatial leakage; methods which suffer from spatial leakage; and finally methods which are immune, but which have had orthogonalisation applied for completeness. AEC - amplitude envelope correlation; PartialCorr - amplitude envelope partial correlation; PLI - phase lag index; wPLI - weighted phase lag index; PLV - phase locking value; Coh - band-averaged coherence; IMC - band-averaged imaginary component of coherency; PCoh - partial coherence; IMPC - imaginary partial coherence; PSI - phase slope index; MI - mutual information of phases; PDC - partial directed coherence.

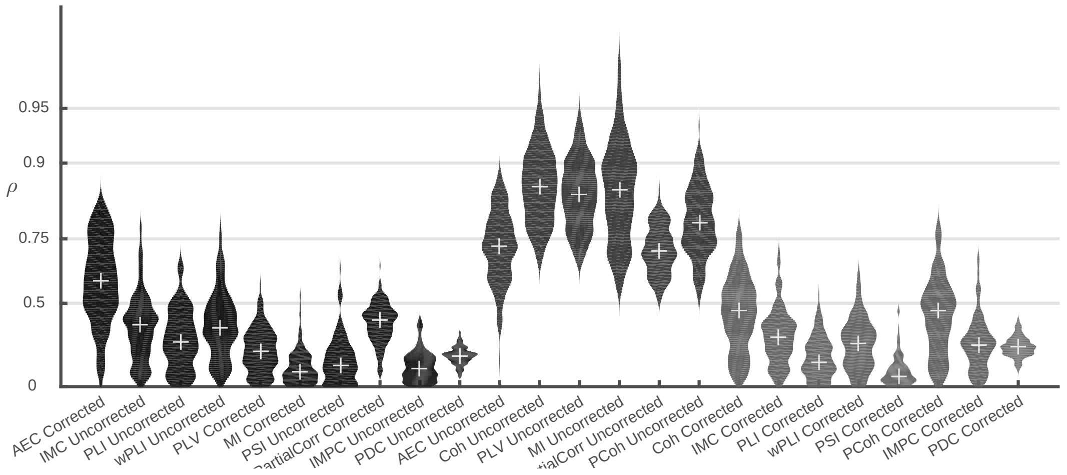

Figure 13: Within-subject consistency of alpha-band (8–13 Hz) network inference. The figure shows correlations between alpha-band network matrices inferred from each of three resting-state sessions from 61 subjects of the HCP dataset. The violin plots show smoothed histograms of the correlations within each subject, the median marked by a white cross. Network estimation methods are ordered to match the figure in the main text: methods immune to or corrected for spatial leakage; methods which suffer from spatial leakage; and finally methods which are immune, but which have had orthogonalisation applied for completeness. AEC - amplitude envelope correlation; PartialCorr - amplitude envelope partial correlation; PLI - phase lag index; wPLI - weighted phase lag index; PLV - phase locking value; Coh - band-averaged coherence; IMC - band-averaged imaginary component of coherency; PCoh - partial coherence; IMPC - imaginary partial coherence; PSI - phase slope index; MI - mutual information of phases; PDC - partial directed coherence.

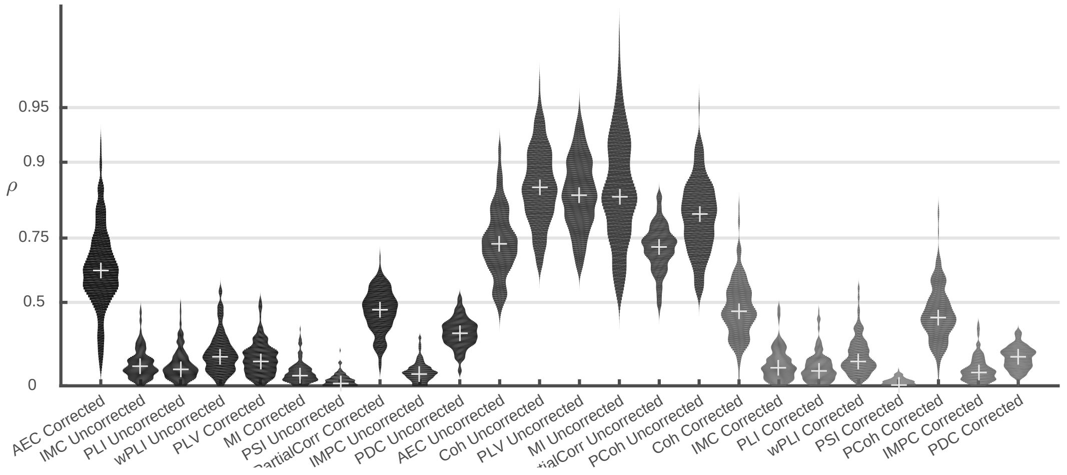

Figure 14: Within-subject consistency of beta-band (13–30 Hz) network inference. The figure shows correlations between beta-band network matrices inferred from each of three resting-state sessions from 61 subjects of the HCP dataset. The violin plots show smoothed histograms of the correlations within each subject, the median marked by a white cross. Network estimation methods are ordered to match the figure in the main text: methods immune to or corrected for spatial leakage; methods which suffer from spatial leakage; and finally methods which are immune, but which have had orthogonalisation applied for completeness. AEC - amplitude envelope correlation; PartialCorr - amplitude envelope partial correlation; PLI - phase lag index; wPLI - weighted phase lag index; PLV - phase locking value; Coh - band-averaged coherence; IMC - band-averaged imaginary component of coherency; PCoh - partial coherence; IMPC - imaginary partial coherence; PSI - phase slope index; MI - mutual information of phases; PDC - partial directed coherence.

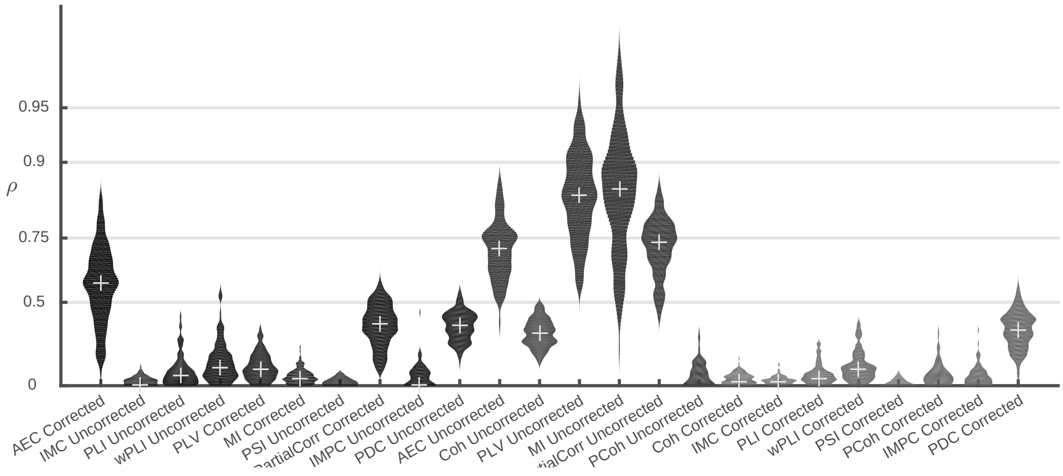

Figure 15: Within-subject consistency of low-beta-band (13–20 Hz) network inference. The figure shows correlations between beta-band network matrices inferred from each of three resting-state sessions from 61 subjects of the HCP dataset. The violin plots show smoothed histograms of the correlations within each subject, the median marked by a white cross. Network estimation methods are ordered to match the figure in the main text: methods immune to or corrected for spatial leakage; methods which suffer from spatial leakage; and finally methods which are immune, but which have had orthogonalisation applied for completeness. AEC - amplitude envelope correlation; PartialCorr - amplitude envelope partial correlation; PLI - phase lag index; wPLI - weighted phase lag index; PLV - phase locking value; Coh - band-averaged coherence; IMC - band-averaged imaginary component of coherency; PCoh - partial coherence; IMPC - imaginary partial coherence; PSI - phase slope index; MI - mutual information of phases; PDC - partial directed coherence.

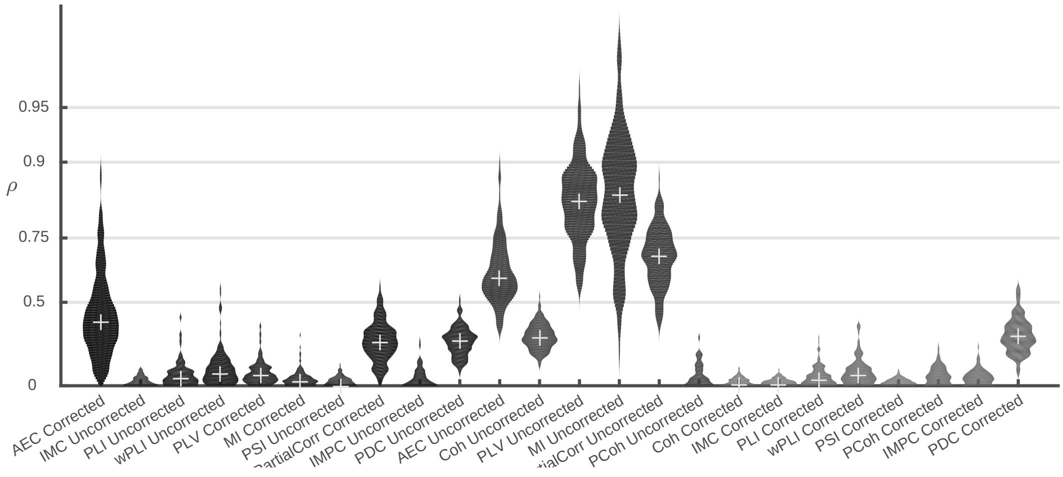

Figure 16: Within-subject consistency of high-beta-band (20–30 Hz) network inference. The figure shows correlations between beta-band network matrices inferred from each of three resting-state sessions from 61 subjects of the HCP dataset. The violin plots show smoothed histograms of the correlations within each subject, the median marked by a white cross. Network estimation methods are ordered to match the figure in the main text: methods immune to or corrected for spatial leakage; methods which suffer from spatial leakage; and finally methods which are immune, but which have had orthogonalisation applied for completeness. AEC - amplitude envelope correlation; PartialCorr - amplitude envelope partial correlation; PLI - phase lag index; wPLI - weighted phase lag index; PLV - phase locking value; Coh - band-averaged coherence; IMC - band-averaged imaginary component of coherency; PCoh - partial coherence; IMPC - imaginary partial coherence; PSI - phase slope index; MI - mutual information of phases; PDC - partial directed coherence.

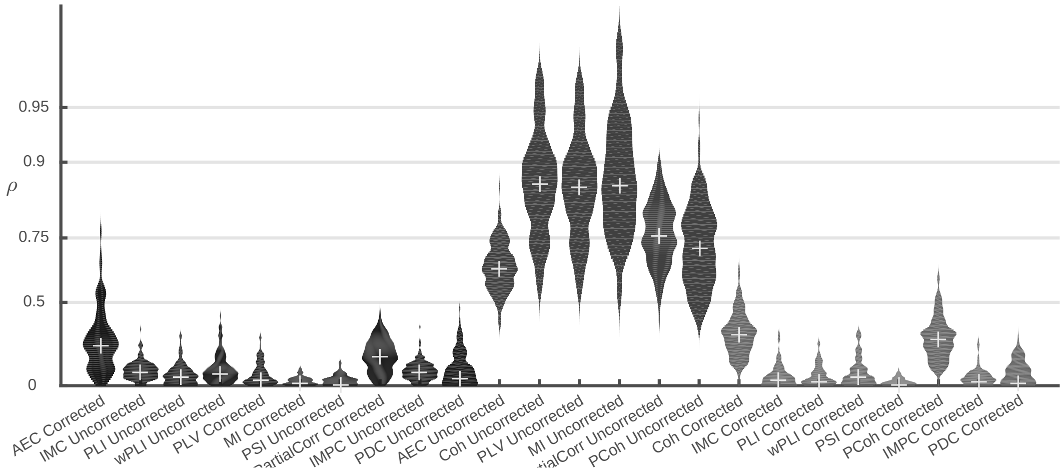

Figure 17: Within-subject consistency of gamma-band (30–48 Hz) network inference. The figure shows correlations between beta-band network matrices inferred from each of three resting-state sessions from 61 subjects of the HCP dataset. The violin plots show smoothed histograms of the correlations within each subject, the median marked by a white cross. Network estimation methods are ordered to match the figure in the main text: methods immune to or corrected for spatial leakage; methods which suffer from spatial leakage; and finally methods which are immune, but which have had orthogonalisation applied for completeness. AEC - amplitude envelope correlation; PartialCorr - amplitude envelope partial correlation; PLI - phase lag index; wPLI - weighted phase lag index; PLV - phase locking value; Coh - band-averaged coherence; IMC - band-averaged imaginary component of coherency; PCoh - partial coherence; IMPC - imaginary partial coherence; PSI - phase slope index; MI - mutual information of phases; PDC - partial directed coherence.

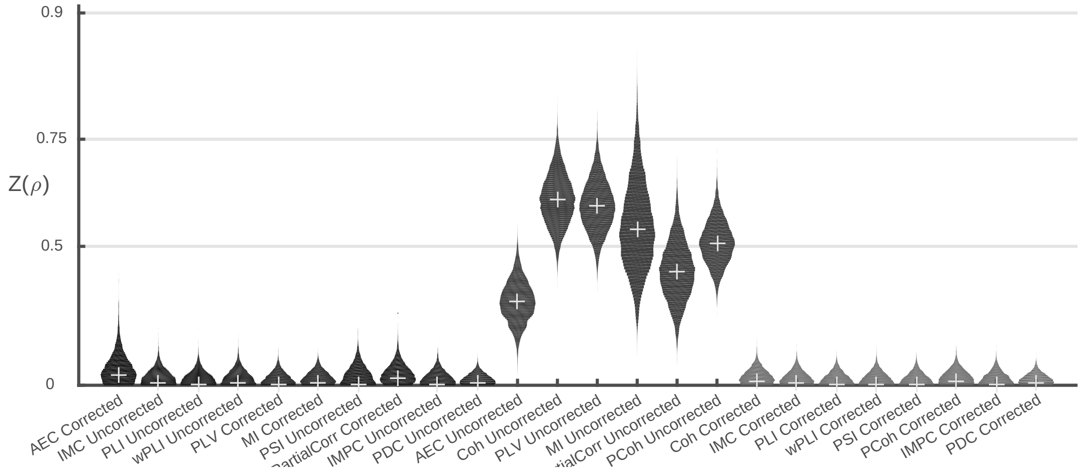

Figure 18: Between-subject consistency of delta-band (1–4 Hz) network inference. The figure shows correlations between delta-band network matrices inferred from 61 subjects of the HCP dataset. The violin plots show smoothed histograms of the correlations within each subject, over all pairings of sessions, the median marked by a white cross. Network estimation methods are ordered to match the figure in the main text: methods immune to or corrected for spatial leakage; methods which suffer from spatial leakage; and finally methods which are immune, but which have had orthogonalisation applied for completeness. AEC - amplitude envelope correlation; PartialCorr - amplitude envelope partial correlation; PLI - phase lag index; wPLI - weighted phase lag index; PLV - phase locking value; Coh - band-averaged coherence; IMC - band-averaged imaginary component of coherency; PCoh - partial coherence; IMPC - imaginary partial coherence; PSI - phase slope index; MI - mutual information of phases; PDC - partial directed coherence.

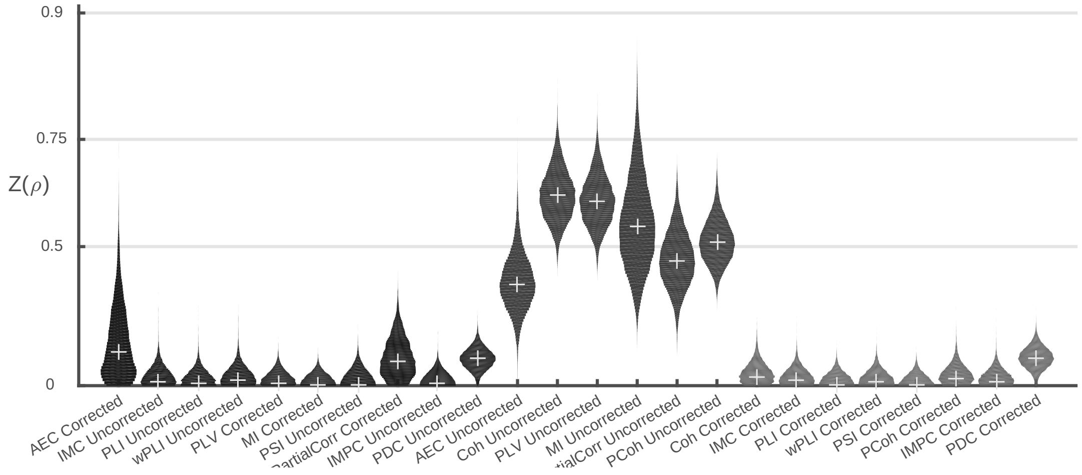

Figure 19: Between-subject consistency of theta-band (4–8 Hz) network inference. The figure shows correlations between theta-band network matrices inferred from 61 subjects of the HCP dataset. The violin plots show smoothed histograms of the correlations within each subject, over all pairings of sessions, the median marked by a white cross. Network estimation methods are ordered to match the figure in the main text: methods immune to or corrected for spatial leakage; methods which suffer from spatial leakage; and finally methods which are immune, but which have had orthogonalisation applied for completeness. AEC - amplitude envelope correlation; PartialCorr - amplitude envelope partial correlation; PLI - phase lag index; wPLI - weighted phase lag index; PLV - phase locking value; Coh - band-averaged coherence; IMC - band-averaged imaginary component of coherency; PCoh - partial coherence; IMPC - imaginary partial coherence; PSI - phase slope index; MI - mutual information of phases; PDC - partial directed coherence.

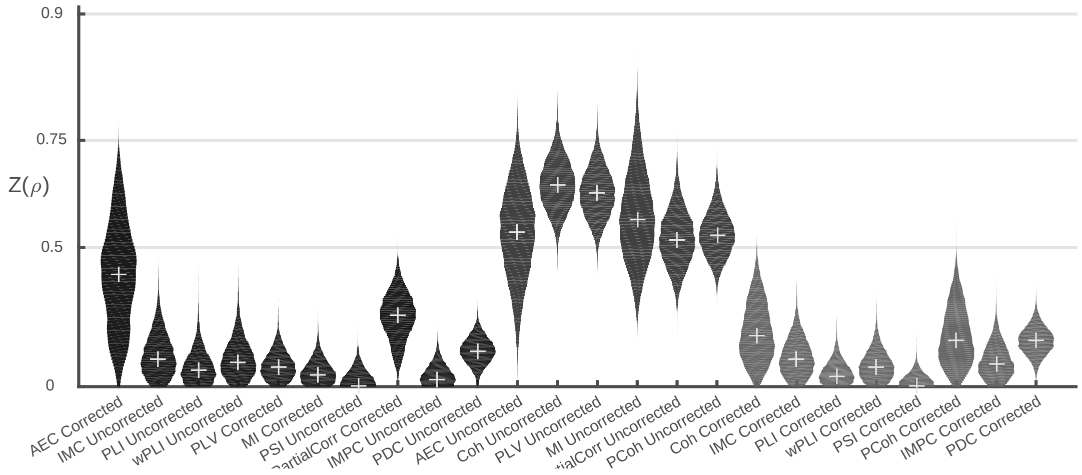

Figure 20: Between-subject consistency of alpha-band (8–13 Hz) network inference. The figure shows correlations between alpha-band network matrices inferred from 61 subjects of the HCP dataset. The violin plots show smoothed histograms of the correlations within each subject, over all pairings of sessions, the median marked by a white cross. Network estimation methods are ordered to match the figure in the main text: methods immune to or corrected for spatial leakage; methods which suffer from spatial leakage; and finally methods which are immune, but which have had orthogonalisation applied for completeness. AEC - amplitude envelope correlation; PartialCorr - amplitude envelope partial correlation; PLI - phase lag index; wPLI - weighted phase lag index; PLV - phase locking value; Coh - band-averaged coherence; IMC - band-averaged imaginary component of coherency; PCoh - partial coherence; IMPC - imaginary partial coherence; PSI - phase slope index; MI - mutual information of phases; PDC - partial directed coherence.

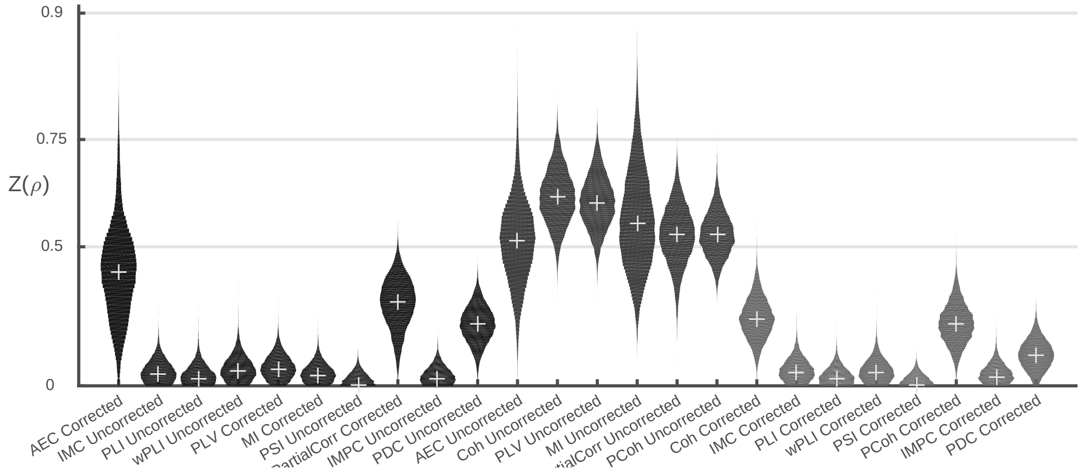

Figure 21: Between-subject consistency of beta-band (13–30 Hz) network inference. The figure shows correlations between beta-band network matrices inferred from 61 subjects of the HCP dataset. The violin plots show smoothed histograms of the correlations within each subject, over all pairings of sessions, the median marked by a white cross. Network estimation methods are ordered to match the figure in the main text: methods immune to or corrected for spatial leakage; methods which suffer from spatial leakage; and finally methods which are immune, but which have had orthogonalisation applied for completeness. AEC - amplitude envelope correlation; PartialCorr - amplitude envelope partial correlation; PLI - phase lag index; wPLI - weighted phase lag index; PLV - phase locking value; Coh - band-averaged coherence; IMC - band-averaged imaginary component of coherency; PCoh - partial coherence; IMPC - imaginary partial coherence; PSI - phase slope index; MI - mutual information of phases; PDC - partial directed coherence.

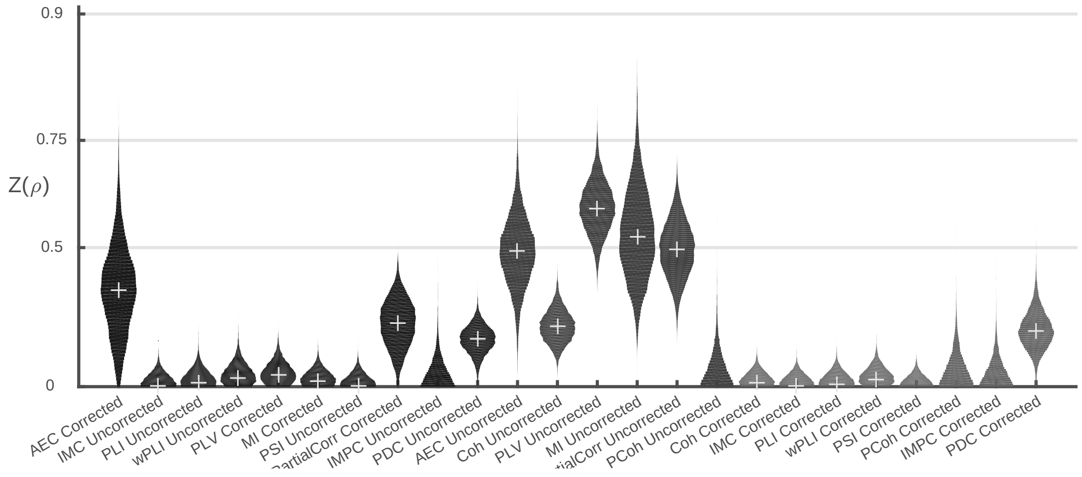

Figure 22: Between-subject consistency of low-beta-band (13–20 Hz) network inference. The figure shows correlations between beta-band network matrices inferred from 61 subjects of the HCP dataset. The violin plots show smoothed histograms of the correlations within each subject, over all pairings of sessions, the median marked by a white cross. Network estimation methods are ordered to match the figure in the main text: methods immune to or corrected for spatial leakage; methods which suffer from spatial leakage; and finally methods which are immune, but which have had orthogonalisation applied for completeness. AEC - amplitude envelope correlation; PartialCorr - amplitude envelope partial correlation; PLI - phase lag index; wPLI - weighted phase lag index; PLV - phase locking value; Coh - band-averaged coherence; IMC - band-averaged imaginary component of coherency; PCoh - partial coherence; IMPC - imaginary partial coherence; PSI - phase slope index; MI - mutual information of phases; PDC - partial directed coherence.

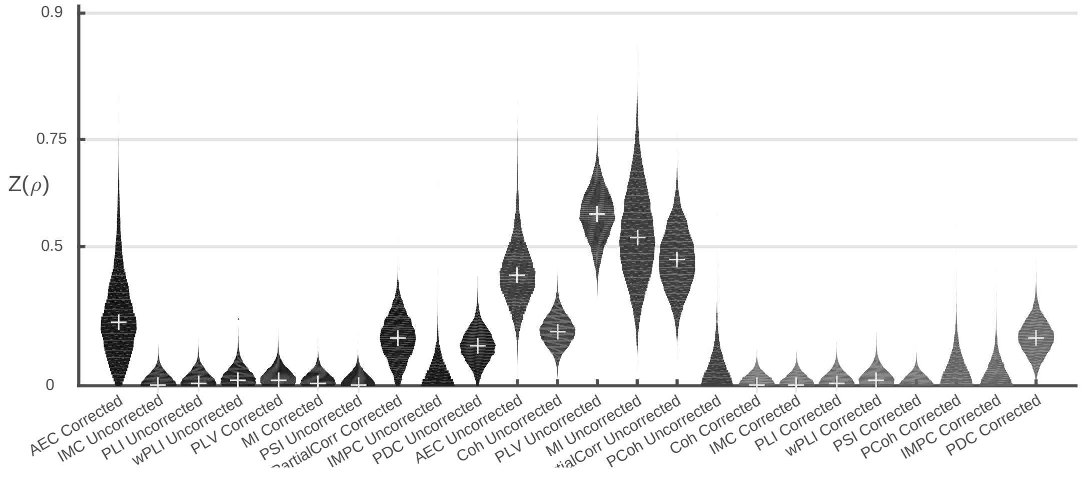

Figure 23: Between-subject consistency of high-beta-band (20–30 Hz) network inference. The figure shows correlations between beta-band network matrices inferred from 61 subjects of the HCP dataset. The violin plots show smoothed histograms of the correlations within each subject, over all pairings of sessions, the median marked by a white cross. Network estimation methods are ordered to match the figure in the main text: methods immune to or corrected for spatial leakage; methods which suffer from spatial leakage; and finally methods which are immune, but which have had orthogonalisation applied for completeness. AEC - amplitude envelope correlation; PartialCorr - amplitude envelope partial correlation; PLI - phase lag index; wPLI - weighted phase lag index; PLV - phase locking value; Coh - band-averaged coherence; IMC - band-averaged imaginary component of coherency; PCoh - partial coherence; IMPC - imaginary partial coherence; PSI - phase slope index; MI - mutual information of phases; PDC - partial directed coherence.

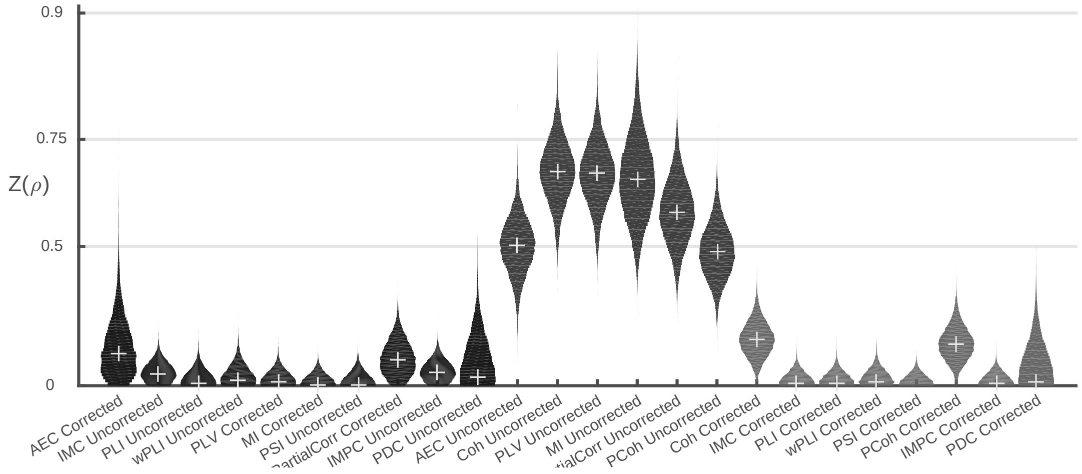

Figure 24: Between-subject consistency of gamma-band (30–48 Hz) network inference. The figure shows correlations between beta-band network matrices inferred from 61 subjects of the HCP dataset. The violin plots show smoothed histograms of the correlations within each subject, over all pairings of sessions, the median marked by a white cross. Network estimation methods are ordered to match the figure in the main text: methods immune to or corrected for spatial leakage; methods which suffer from spatial leakage; and finally methods which are immune, but which have had orthogonalisation applied for completeness. AEC - amplitude envelope correlation; PartialCorr - amplitude envelope partial correlation; PLI - phase lag index; wPLI - weighted phase lag index; PLV - phase locking value; Coh - band-averaged coherence; IMC - band-averaged imaginary component of coherency; PCoh - partial coherence; IMPC - imaginary partial coherence; PSI - phase slope index; MI - mutual information of phases; PDC - partial directed coherence.

8. SI2: Full results using 2-second windows for phase and spectrum estimation

8.1. Split-half reliability

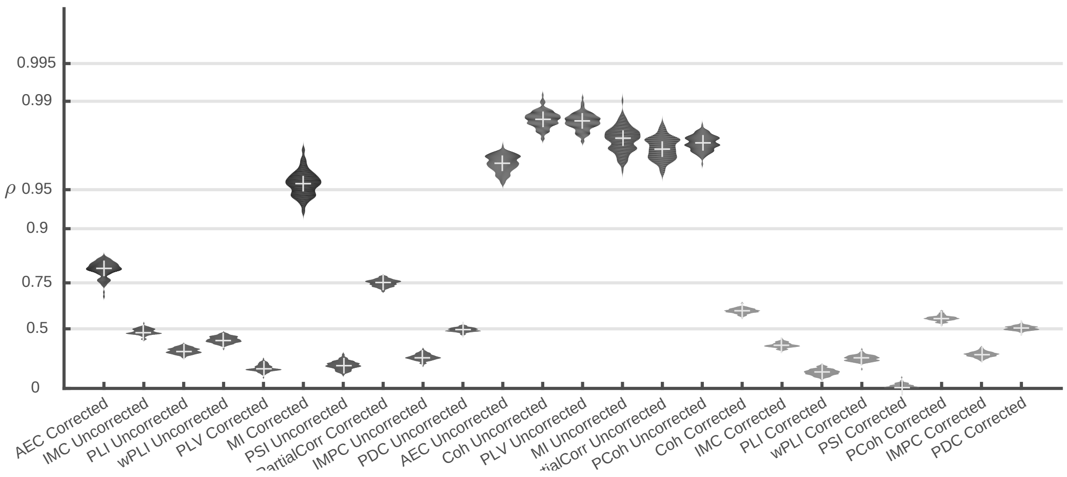

Figure 25: Stability of delta-band (1–4 Hz) group-level inference. The figure displays correlations between network matrices inferred from separate halves of the HCP dataset, using resting-state recordings in the delta band. The dataset was randomly partitioned in half 100 times, and the distribution of correlations between the network edge strengths in each half produced over the bootstrapped samples. The violin plots show smoothed histograms of these distributions, the median marked by a white cross. Network estimation methods are ordered to match the figure in the main text: methods immune to or corrected for spatial leakage; methods which suffer from spatial leakage; and finally methods which are immune, but which have had orthogonalisation applied for completeness. AEC - amplitude envelope correlation; PartialCorr - amplitude envelope partial correlation; PLI - phase lag index; wPLI - weighted phase lag index; PLV - phase locking value; Coh - band-averaged coherence; IMC - band-averaged imaginary component of coherency; PCoh - partial coherence; IMPC - imaginary partial coherence; PSI - phase slope index; MI - mutual information of phases; PDC - partial directed coherence.

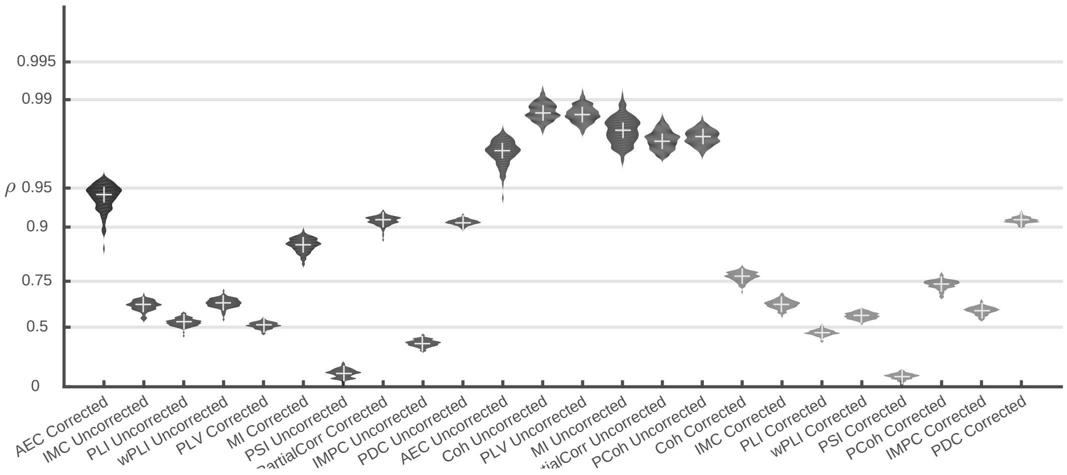

Figure 26: Stability of theta-band (4–8 Hz) group-level inference. The figure displays correlations between network matrices inferred from separate halves of the HCP dataset, using resting-state recordings in the theta band. The dataset was randomly partitioned in half 100 times, and the distribution of correlations between the network edge strengths in each half produced over the bootstrapped samples. The violin plots show smoothed histograms of these distributions, the median marked by a white cross. Network estimation methods are ordered to match the figure in the main text: methods immune to or corrected for spatial leakage; methods which suffer from spatial leakage; and finally methods which are immune, but which have had orthogonalisation applied for completeness. AEC - amplitude envelope correlation; PartialCorr - amplitude envelope partial correlation; PLI - phase lag index; wPLI - weighted phase lag index; PLV - phase locking value; Coh - band-averaged coherence; IMC - band-averaged imaginary component of coherency; PCoh - partial coherence; IMPC - imaginary partial coherence; PSI - phase slope index; MI - mutual information of phases; PDC - partial directed coherence.

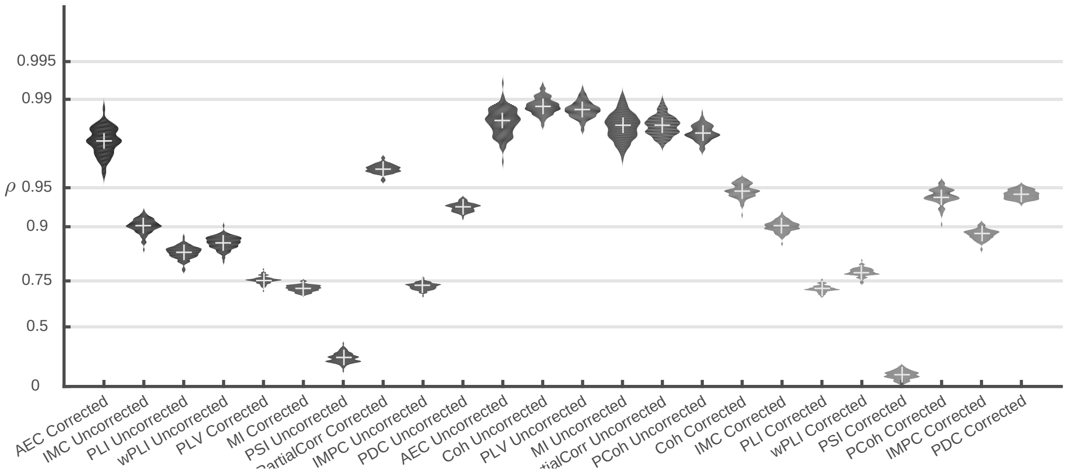

Figure 27: Stability of alpha-band (8–13 Hz) group-level inference. The figure displays correlations between network matrices inferred from separate halves of the HCP dataset, using resting-state recordings in the theta band. The dataset was randomly partitioned in half 100 times, and the distribution of correlations between the network edge strengths in each half produced over the bootstrapped samples. The violin plots show smoothed histograms of these distributions, the median marked by a white cross. Network estimation methods are ordered to match the figure in the main text: methods immune to or corrected for spatial leakage; methods which suffer from spatial leakage; and finally methods which are immune, but which have had orthogonalisation applied for completeness. AEC - amplitude envelope correlation; PartialCorr - amplitude envelope partial correlation; PLI - phase lag index; wPLI - weighted phase lag index; PLV - phase locking value; Coh - band-averaged coherence; IMC - band-averaged imaginary component of coherency; PCoh - partial coherence; IMPC - imaginary partial coherence; PSI - phase slope index; MI - mutual information of phases; PDC - partial directed coherence.

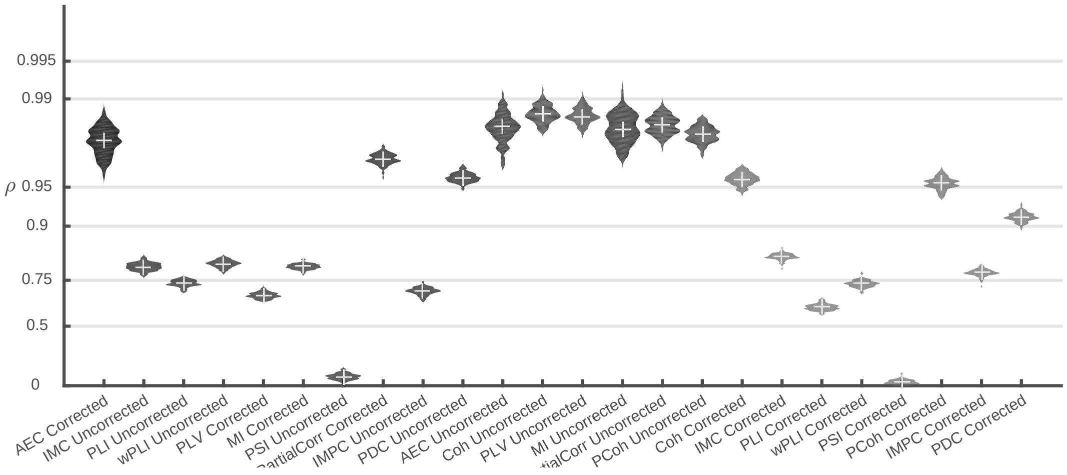

Figure 28: Stability of beta-band (13–30 Hz) group-level inference. The figure displays correlations between network matrices inferred from separate halves of the HCP dataset, using resting-state recordings in the beta band. The dataset was randomly partitioned in half 100 times, and the distribution of correlations between the network edge strengths in each half produced over the bootstrapped samples. The violin plots show smoothed histograms of these distributions, the median marked by a white cross. Network estimation methods are ordered to match the figure in the main text: methods immune to or corrected for spatial leakage; methods which suffer from spatial leakage; and finally methods which are immune, but which have had orthogonalisation applied for completeness. AEC - amplitude envelope correlation; PartialCorr - amplitude envelope partial correlation; PLI - phase lag index; wPLI - weighted phase lag index; PLV - phase locking value; Coh - band-averaged coherence; IMC - band-averaged imaginary component of coherency; PCoh - partial coherence; IMPC - imaginary partial coherence; PSI - phase slope index; MI - mutual information of phases; PDC - partial directed coherence.

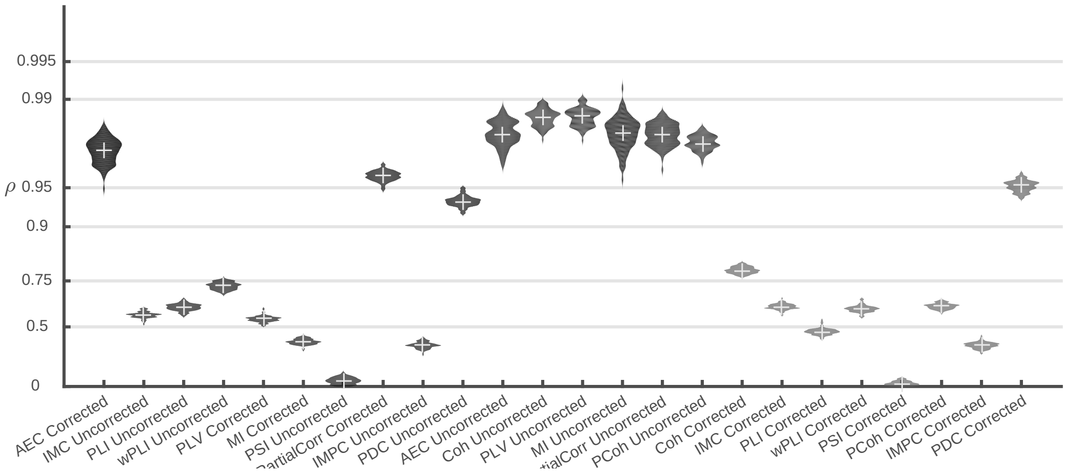

Figure 29: Stability of low-beta-band (13–20 Hz) group-level inference. The figure displays correlations between network matrices inferred from separate halves of the HCP dataset, using resting-state recordings in the beta band. The dataset was randomly partitioned in half 100 times, and the distribution of correlations between the network edge strengths in each half produced over the bootstrapped samples. The violin plots show smoothed histograms of these distributions, the median marked by a white cross. Network estimation methods are ordered to match the figure in the main text: methods immune to or corrected for spatial leakage; methods which suffer from spatial leakage; and finally methods which are immune, but which have had orthogonalisation applied for completeness. AEC - amplitude envelope correlation; PartialCorr - amplitude envelope partial correlation; PLI - phase lag index; wPLI - weighted phase lag index; PLV - phase locking value; Coh - band-averaged coherence; IMC - band-averaged imaginary component of coherency; PCoh - partial coherence; IMPC - imaginary partial coherence; PSI - phase slope index; MI - mutual information of phases; PDC - partial directed coherence.

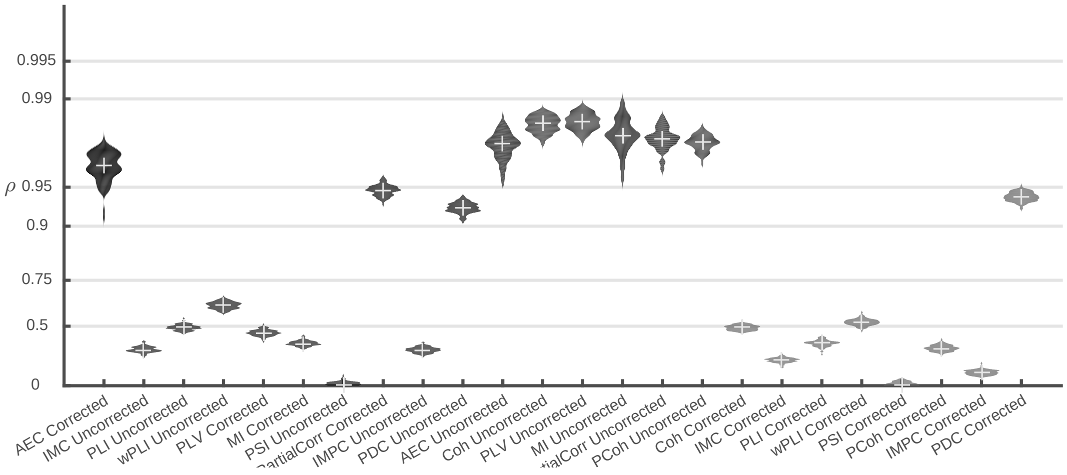

Figure 30: Stability of high-beta-band (20–30 Hz) group-level inference. The figure displays correlations between network matrices inferred from separate halves of the HCP dataset, using resting-state recordings in the beta band. The dataset was randomly partitioned in half 100 times, and the distribution of correlations between the network edge strengths in each half produced over the bootstrapped samples. The violin plots show smoothed histograms of these distributions, the median marked by a white cross. Network estimation methods are ordered to match the figure in the main text: methods immune to or corrected for spatial leakage; methods which suffer from spatial leakage; and finally methods which are immune, but which have had orthogonalisation applied for completeness. AEC - amplitude envelope correlation; PartialCorr - amplitude envelope partial correlation; PLI - phase lag index; wPLI - weighted phase lag index; PLV - phase locking value; Coh - band-averaged coherence; IMC - band-averaged imaginary component of coherency; PCoh - partial coherence; IMPC - imaginary partial coherence; PSI - phase slope index; MI - mutual information of phases; PDC - partial directed coherence.

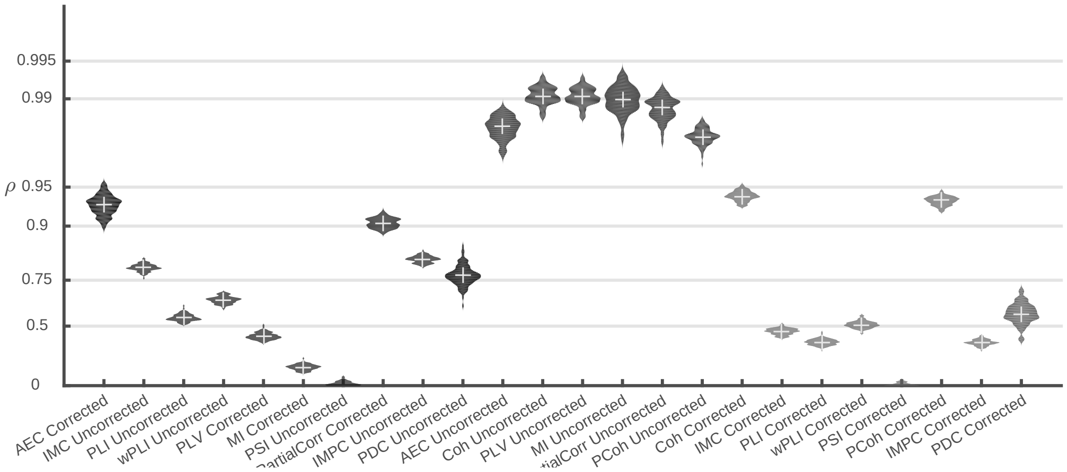

Figure 31: Stability of gamma-band (30–48 Hz) group-level inference. The figure displays correlations between network matrices inferred from separate halves of the HCP dataset, using resting-state recordings in the beta band. The dataset was randomly partitioned in half 100 times, and the distribution of correlations between the network edge strengths in each half produced over the bootstrapped samples. The violin plots show smoothed histograms of these distributions, the median marked by a white cross. Network estimation methods are ordered to match the figure in the main text: methods immune to or corrected for spatial leakage; methods which suffer from spatial leakage; and finally methods which are immune, but which have had orthogonalisation applied for completeness. AEC - amplitude envelope correlation; PartialCorr - amplitude envelope partial correlation; PLI - phase lag index; wPLI - weighted phase lag index; PLV - phase locking value; Coh - band-averaged coherence; IMC - band-averaged imaginary component of coherency; PCoh - partial coherence; IMPC - imaginary partial coherence; PSI - phase slope index; MI - mutual information of phases; PDC - partial directed coherence.

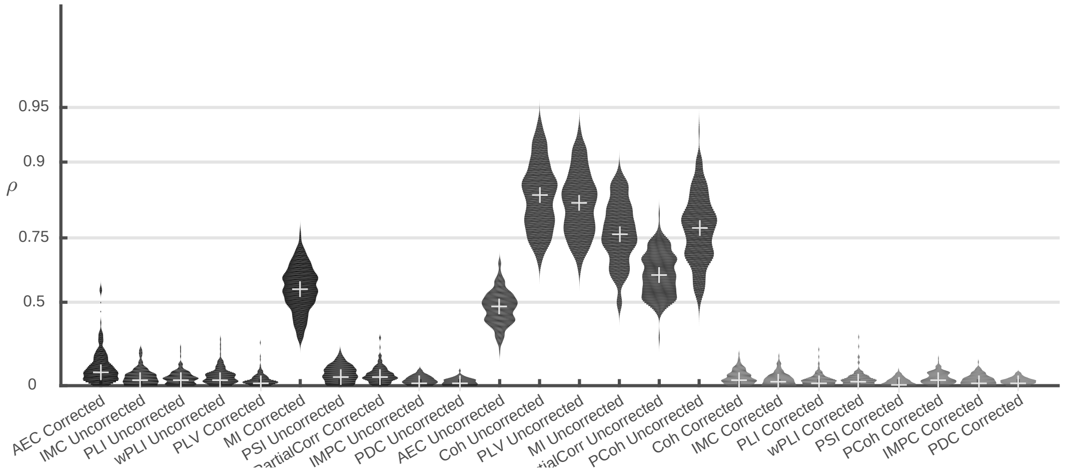

Figure 32: Within-subject consistency of delta-band (1–4 Hz) network inference. The figure shows correlations between delta-band network matrices inferred from each of three resting-state sessions from 61 subjects of the HCP dataset. The violin plots show smoothed histograms of the correlations within each subject, the median marked by a white cross. Network estimation methods are ordered to match the figure in the main text: methods immune to or corrected for spatial leakage; methods which suffer from spatial leakage; and finally methods which are immune, but which have had orthogonalisation applied for completeness. AEC - amplitude envelope correlation; PartialCorr - amplitude envelope partial correlation; PLI - phase lag index; wPLI - weighted phase lag index; PLV - phase locking value; Coh - band-averaged coherence; IMC - band-averaged imaginary component of coherency; PCoh - partial coherence; IMPC - imaginary partial coherence; PSI - phase slope index; MI - mutual information of phases; PDC - partial directed coherence.

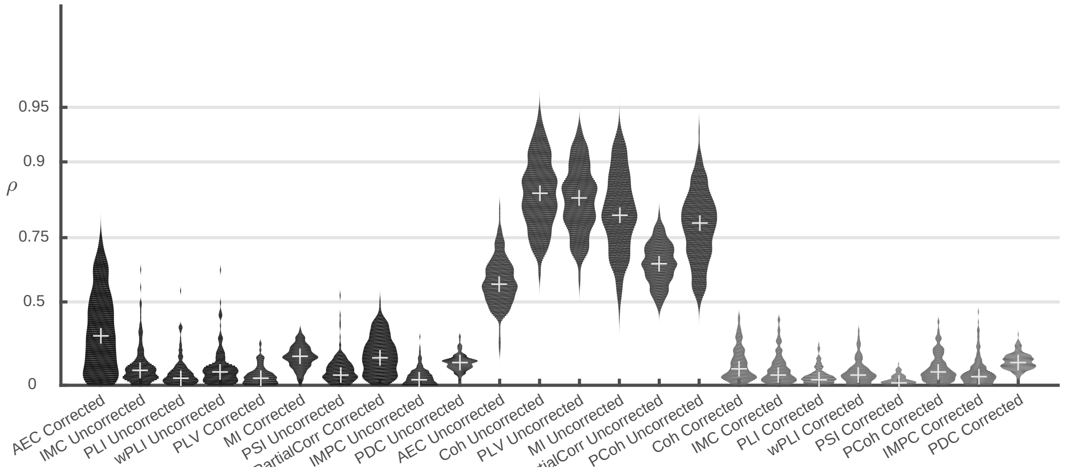

Figure 33: Within-subject consistency of theta-band (4–8 Hz) network inference. The figure shows correlations between theta-band network matrices inferred from each of three resting-state sessions from 61 subjects of the HCP dataset. The violin plots show smoothed histograms of the correlations within each subject, the median marked by a white cross. Network estimation methods are ordered to match the figure in the main text: methods immune to or corrected for spatial leakage; methods which suffer from spatial leakage; and finally methods which are immune, but which have had orthogonalisation applied for completeness. AEC - amplitude envelope correlation; PartialCorr - amplitude envelope partial correlation; PLI - phase lag index; wPLI - weighted phase lag index; PLV - phase locking value; Coh - band-averaged coherence; IMC - band-averaged imaginary component of coherency; PCoh - partial coherence; IMPC - imaginary partial coherence; PSI - phase slope index; MI - mutual information of phases; PDC - partial directed coherence.

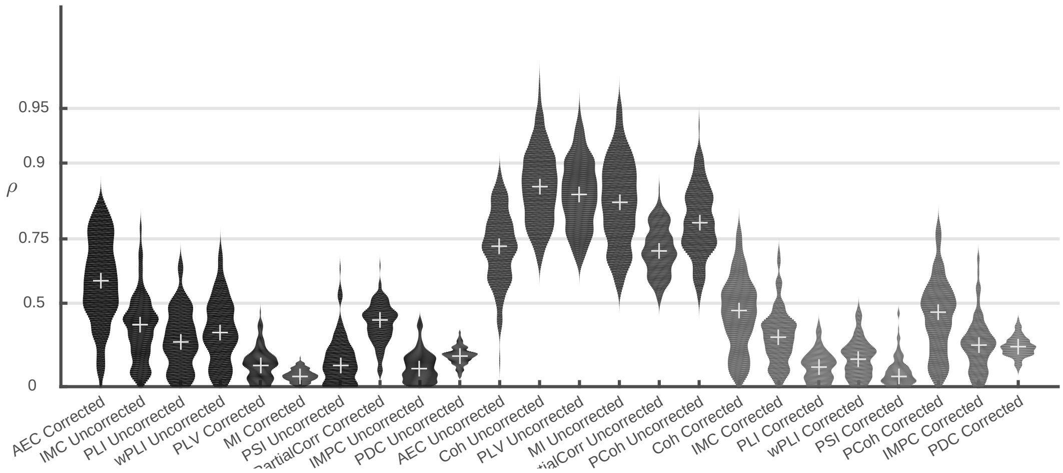

Figure 34: Within-subject consistency of alpha-band (8–13 Hz) network inference. The figure shows correlations between alpha-band network matrices inferred from each of three resting-state sessions from 61 subjects of the HCP dataset. The violin plots show smoothed histograms of the correlations within each subject, the median marked by a white cross. Network estimation methods are ordered to match the figure in the main text: methods immune to or corrected for spatial leakage; methods which suffer from spatial leakage; and finally methods which are immune, but which have had orthogonalisation applied for completeness. AEC - amplitude envelope correlation; PartialCorr - amplitude envelope partial correlation; PLI - phase lag index; wPLI - weighted phase lag index; PLV - phase locking value; Coh - band-averaged coherence; IMC - band-averaged imaginary component of coherency; PCoh - partial coherence; IMPC - imaginary partial coherence; PSI - phase slope index; MI - mutual information of phases; PDC - partial directed coherence.

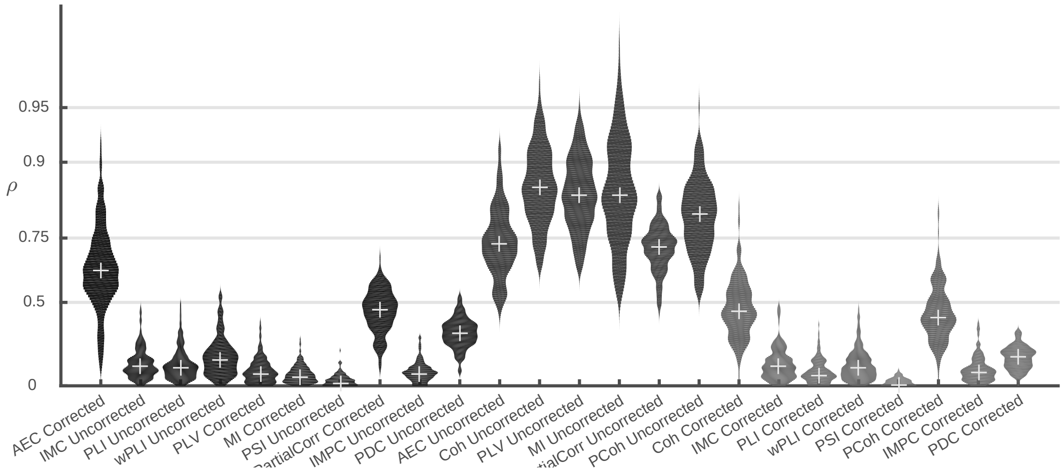

Figure 35: Within-subject consistency of beta-band (13–30 Hz) network inference. The figure shows correlations between beta-band network matrices inferred from each of three resting-state sessions from 61 subjects of the HCP dataset. The violin plots show smoothed histograms of the correlations within each subject, the median marked by a white cross. Network estimation methods are ordered to match the figure in the main text: methods immune to or corrected for spatial leakage; methods which suffer from spatial leakage; and finally methods which are immune, but which have had orthogonalisation applied for completeness. AEC - amplitude envelope correlation; PartialCorr - amplitude envelope partial correlation; PLI - phase lag index; wPLI - weighted phase lag index; PLV - phase locking value; Coh - band-averaged coherence; IMC - band-averaged imaginary component of coherency; PCoh - partial coherence; IMPC - imaginary partial coherence; PSI - phase slope index; MI - mutual information of phases; PDC - partial directed coherence.

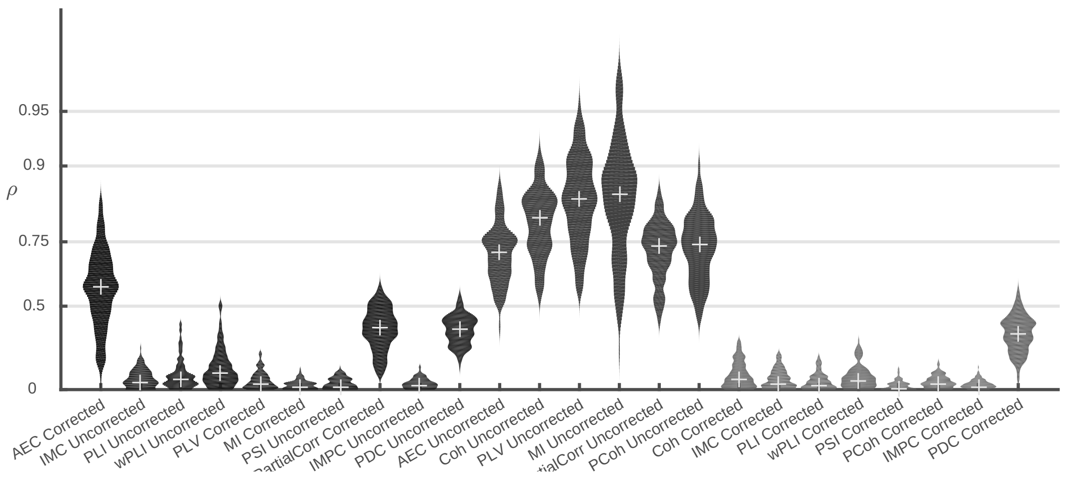

Figure 36: Within-subject consistency of low-beta-band (13–20 Hz) network inference. The figure shows correlations between beta-band network matrices inferred from each of three resting-state sessions from 61 subjects of the HCP dataset. The violin plots show smoothed histograms of the correlations within each subject, the median marked by a white cross. Network estimation methods are ordered to match the figure in the main text: methods immune to or corrected for spatial leakage; methods which suffer from spatial leakage; and finally methods which are immune, but which have had orthogonalisation applied for completeness. AEC - amplitude envelope correlation; PartialCorr - amplitude envelope partial correlation; PLI - phase lag index; wPLI - weighted phase lag index; PLV - phase locking value; Coh - band-averaged coherence; IMC - band-averaged imaginary component of coherency; PCoh - partial coherence; IMPC - imaginary partial coherence; PSI - phase slope index; MI - mutual information of phases; PDC - partial directed coherence.

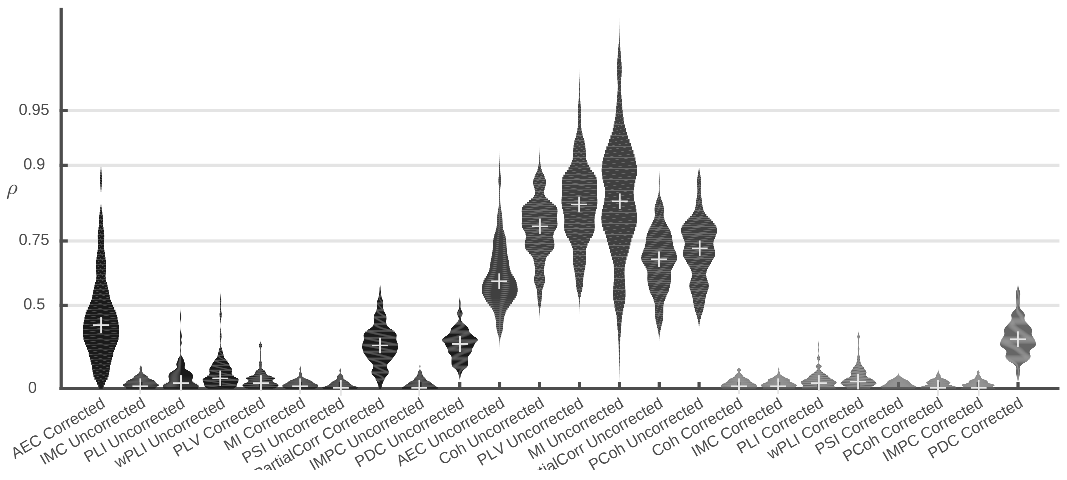

Figure 37: Within-subject consistency of high-beta-band (20–30 Hz) network inference. The figure shows correlations between beta-band network matrices inferred from each of three resting-state sessions from 61 subjects of the HCP dataset. The violin plots show smoothed histograms of the correlations within each subject, the median marked by a white cross. Network estimation methods are ordered to match the figure in the main text: methods immune to or corrected for spatial leakage; methods which suffer from spatial leakage; and finally methods which are immune, but which have had orthogonalisation applied for completeness. AEC - amplitude envelope correlation; PartialCorr - amplitude envelope partial correlation; PLI - phase lag index; wPLI - weighted phase lag index; PLV - phase locking value; Coh - band-averaged coherence; IMC - band-averaged imaginary component of coherency; PCoh - partial coherence; IMPC - imaginary partial coherence; PSI - phase slope index; MI - mutual information of phases; PDC - partial directed coherence.

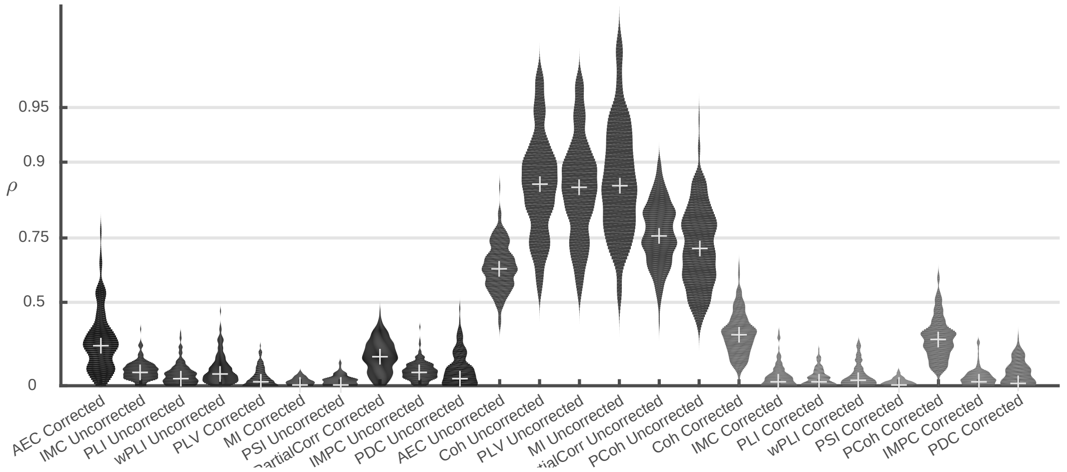

Figure 38: Within-subject consistency of gamma-band (30–48 Hz) network inference. The figure shows correlations between beta-band network matrices inferred from each of three resting-state sessions from 61 subjects of the HCP dataset. The violin plots show smoothed histograms of the correlations within each subject, the median marked by a white cross. Network estimation methods are ordered to match the figure in the main text: methods immune to or corrected for spatial leakage; methods which suffer from spatial leakage; and finally methods which are immune, but which have had orthogonalisation applied for completeness. AEC - amplitude envelope correlation; PartialCorr - amplitude envelope partial correlation; PLI - phase lag index; wPLI - weighted phase lag index; PLV - phase locking value; Coh - band-averaged coherence; IMC - band-averaged imaginary component of coherency; PCoh - partial coherence; IMPC - imaginary partial coherence; PSI - phase slope index; MI - mutual information of phases; PDC - partial directed coherence.

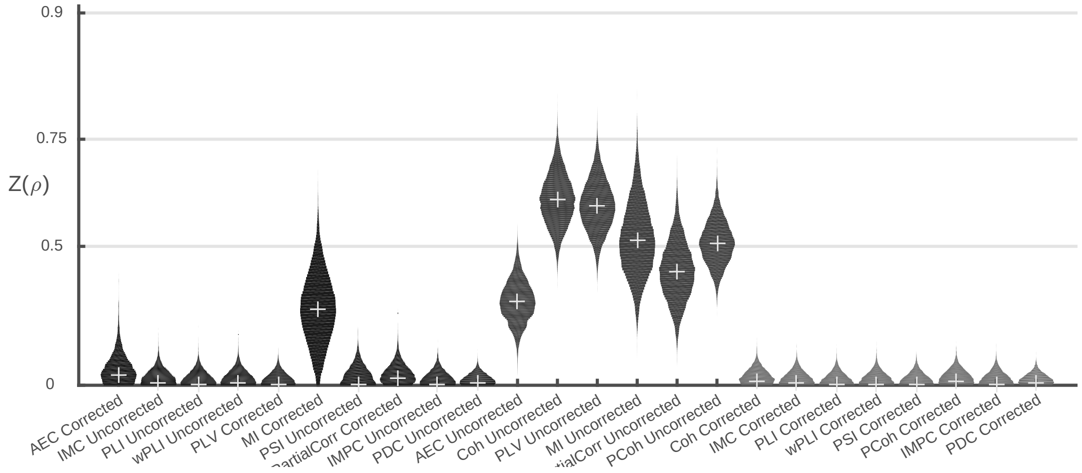

Figure 39: Between-subject consistency of delta-band (1–4 Hz) network inference. The figure shows correlations between delta-band network matrices inferred from 61 subjects of the HCP dataset. The violin plots show smoothed histograms of the correlations within each subject, over all pairings of sessions, the median marked by a white cross. Network estimation methods are ordered to match the figure in the main text: methods immune to or corrected for spatial leakage; methods which suffer from spatial leakage; and finally methods which are immune, but which have had orthogonalisation applied for completeness. AEC - amplitude envelope correlation; PartialCorr - amplitude envelope partial correlation; PLI - phase lag index; wPLI - weighted phase lag index; PLV - phase locking value; Coh - band-averaged coherence; IMC - band-averaged imaginary component of coherency; PCoh - partial coherence; IMPC - imaginary partial coherence; PSI - phase slope index; MI - mutual information of phases; PDC - partial directed coherence.

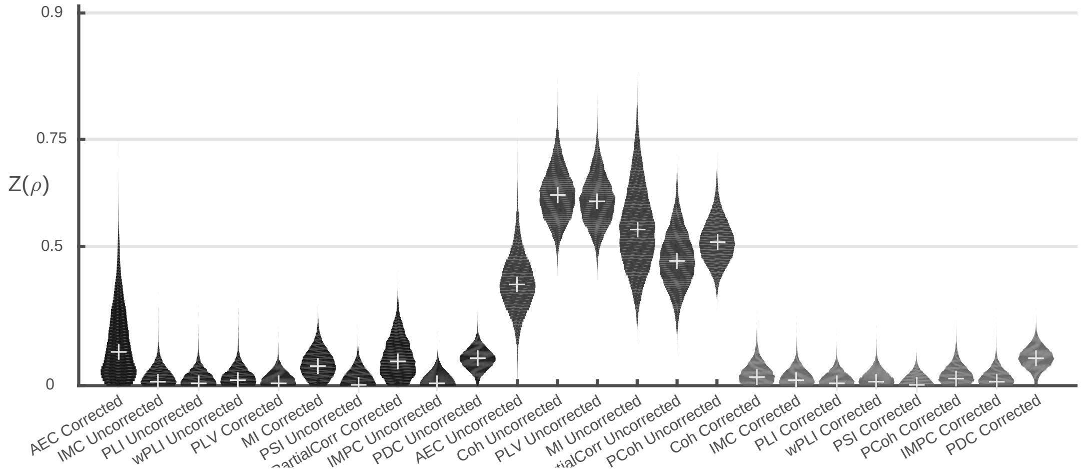

Figure 40: Between-subject consistency of theta-band (4–8 Hz) network inference. The figure shows correlations between theta-band network matrices inferred from 61 subjects of the HCP dataset. The violin plots show smoothed histograms of the correlations within each subject, over all pairings of sessions, the median marked by a white cross. Network estimation methods are ordered to match the figure in the main text: methods immune to or corrected for spatial leakage; methods which suffer from spatial leakage; and finally methods which are immune, but which have had orthogonalisation applied for completeness. AEC - amplitude envelope correlation; PartialCorr - amplitude envelope partial correlation; PLI - phase lag index; wPLI - weighted phase lag index; PLV - phase locking value; Coh - band-averaged coherence; IMC - band-averaged imaginary component of coherency; PCoh - partial coherence; IMPC - imaginary partial coherence; PSI - phase slope index; MI - mutual information of phases; PDC - partial directed coherence.

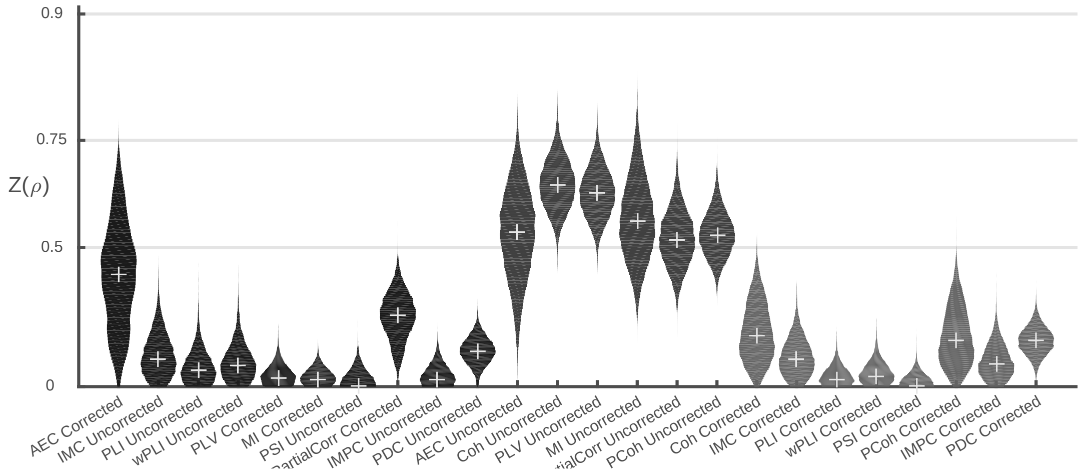

Figure 41: Between-subject consistency of alpha-band (8–13 Hz) network inference. The figure shows correlations between alpha-band network matrices inferred from 61 subjects of the HCP dataset. The violin plots show smoothed histograms of the correlations within each subject, over all pairings of sessions, the median marked by a white cross. Network estimation methods are ordered to match the figure in the main text: methods immune to or corrected for spatial leakage; methods which suffer from spatial leakage; and finally methods which are immune, but which have had orthogonalisation applied for completeness. AEC - amplitude envelope correlation; PartialCorr - amplitude envelope partial correlation; PLI - phase lag index; wPLI - weighted phase lag index; PLV - phase locking value; Coh - band-averaged coherence; IMC - band-averaged imaginary component of coherency; PCoh - partial coherence; IMPC - imaginary partial coherence; PSI - phase slope index; MI - mutual information of phases; PDC - partial directed coherence.

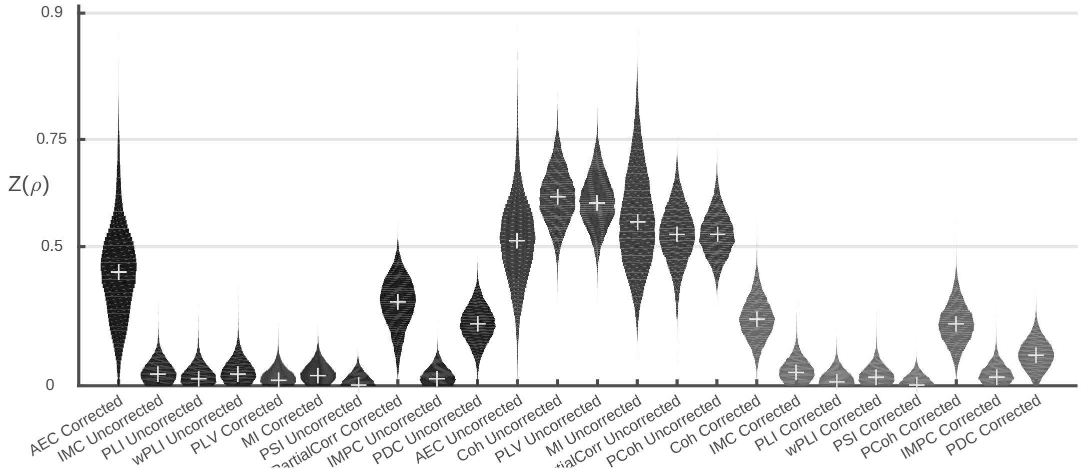

Figure 42: Between-subject consistency of beta-band (13–30 Hz) network inference. The figure shows correlations between beta-band network matrices inferred from 61 subjects of the HCP dataset. The violin plots show smoothed histograms of the correlations within each subject, over all pairings of sessions, the median marked by a white cross. Network estimation methods are ordered to match the figure in the main text: methods immune to or corrected for spatial leakage; methods which suffer from spatial leakage; and finally methods which are immune, but which have had orthogonalisation applied for completeness. AEC - amplitude envelope correlation; PartialCorr - amplitude envelope partial correlation; PLI - phase lag index; wPLI - weighted phase lag index; PLV - phase locking value; Coh - band-averaged coherence; IMC - band-averaged imaginary component of coherency; PCoh - partial coherence; IMPC - imaginary partial coherence; PSI - phase slope index; MI - mutual information of phases; PDC - partial directed coherence.

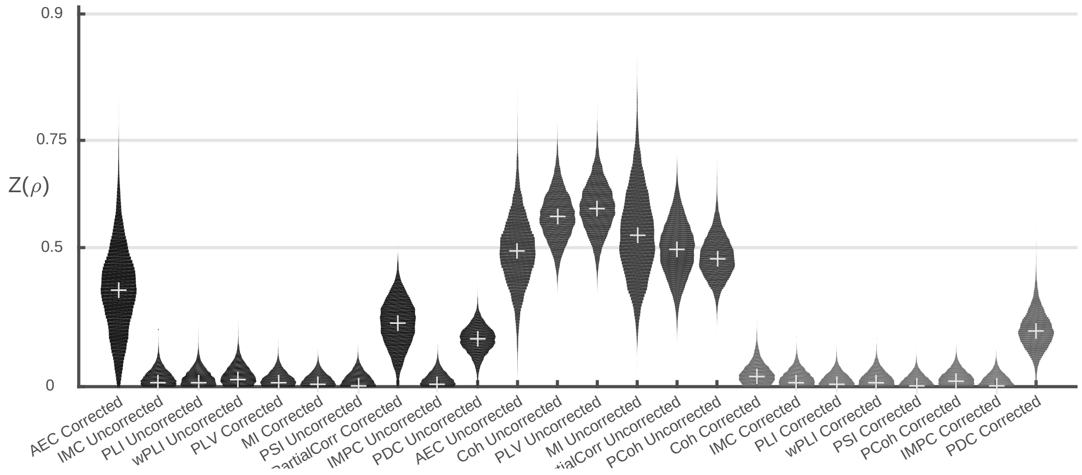

Figure 43: Between-subject consistency of low-beta-band (13–20 Hz) network inference. The figure shows correlations between beta-band network matrices inferred from 61 subjects of the HCP dataset. The violin plots show smoothed histograms of the correlations within each subject, over all pairings of sessions, the median marked by a white cross. Network estimation methods are ordered to match the figure in the main text: methods immune to or corrected for spatial leakage; methods which suffer from spatial leakage; and finally methods which are immune, but which have had orthogonalisation applied for completeness. AEC - amplitude envelope correlation; PartialCorr - amplitude envelope partial correlation; PLI - phase lag index; wPLI - weighted phase lag index; PLV - phase locking value; Coh - band-averaged coherence; IMC - band-averaged imaginary component of coherency; PCoh - partial coherence; IMPC - imaginary partial coherence; PSI - phase slope index; MI - mutual information of phases; PDC - partial directed coherence.

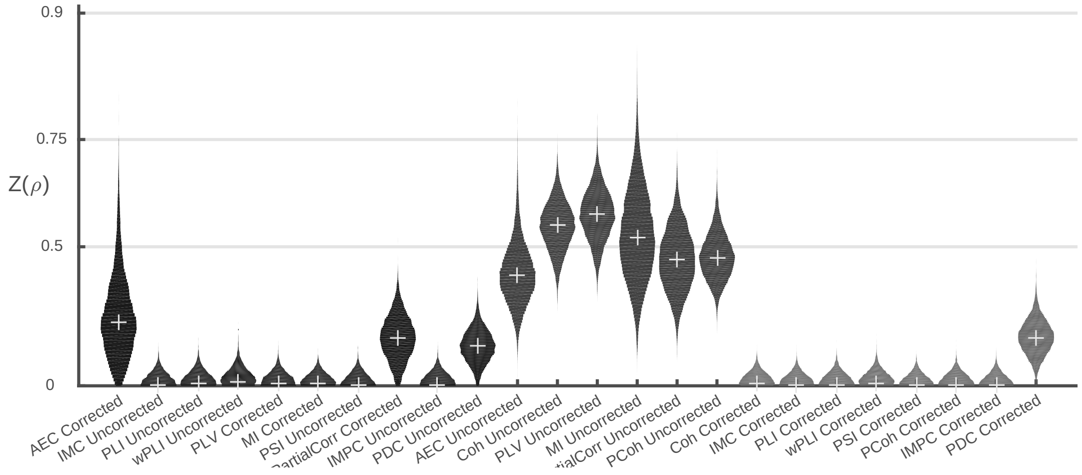

Figure 44: Between-subject consistency of high-beta-band (20–30 Hz) network inference. The figure shows correlations between beta-band network matrices inferred from 61 subjects of the HCP dataset. The violin plots show smoothed histograms of the correlations within each subject, over all pairings of sessions, the median marked by a white cross. Network estimation methods are ordered to match the figure in the main text: methods immune to or corrected for spatial leakage; methods which suffer from spatial leakage; and finally methods which are immune, but which have had orthogonalisation applied for completeness. AEC - amplitude envelope correlation; PartialCorr - amplitude envelope partial correlation; PLI - phase lag index; wPLI - weighted phase lag index; PLV - phase locking value; Coh - band-averaged coherence; IMC - band-averaged imaginary component of coherency; PCoh - partial coherence; IMPC - imaginary partial coherence; PSI - phase slope index; MI - mutual information of phases; PDC - partial directed coherence.

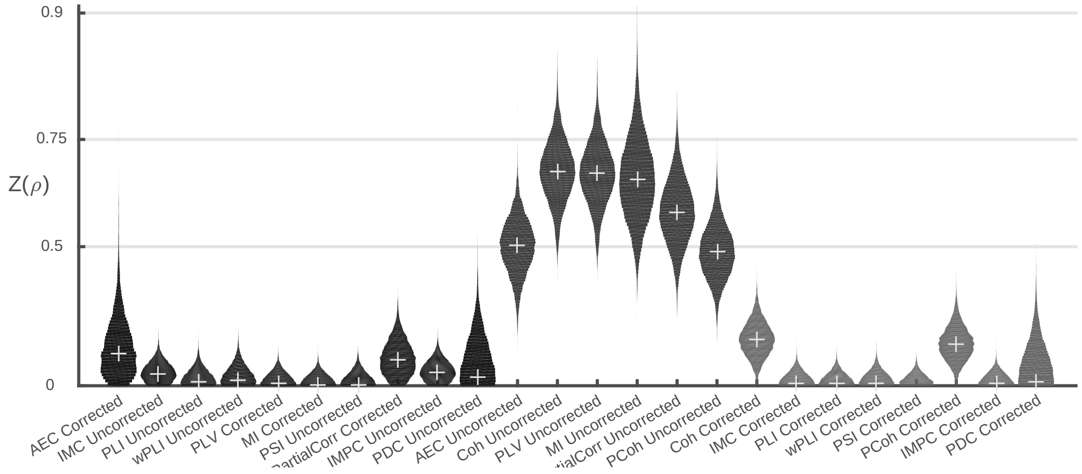

Figure 45: Between-subject consistency of gamma-band (30–48 Hz) network inference. The figure shows correlations between beta-band network matrices inferred from 61 subjects of the HCP dataset. The violin plots show smoothed histograms of the correlations within each subject, over all pairings of sessions, the median marked by a white cross. Network estimation methods are ordered to match the figure in the main text: methods immune to or corrected for spatial leakage; methods which suffer from spatial leakage; and finally methods which are immune, but which have had orthogonalisation applied for completeness. AEC - amplitude envelope correlation; PartialCorr - amplitude envelope partial correlation; PLI - phase lag index; wPLI - weighted phase lag index; PLV - phase locking value; Coh - band-averaged coherence; IMC - band-averaged imaginary component of coherency; PCoh - partial coherence; IMPC - imaginary partial coherence; PSI - phase slope index; MI - mutual information of phases; PDC - partial directed coherence.
